# Disparities in adult critical care resources across Pakistan: findings from a national survey and assessment using a novel scoring system

**DOI:** 10.1186/s13054-022-04046-5

**Published:** 2022-07-11

**Authors:** Mustafa Ali Khan, Hamna Shahbaz, Ali Aahil Noorali, Anam Noor Ehsan, Mareeha Zaki, Fahham Asghar, Mohammed Moizul Hassan, Haroon Muhammad Arshad, Muhammad Sohaib, Muhammad Ali Asghar, Muhammad Faisal Khan, Amber Sabeen, Masooma Aqeel, Muhammad Haroon Khan, Tahir Munir, Syed Kashif Amin, Huba Atiq, Adil Hussain Haider, Zainab Samad, Asad Latif

**Affiliations:** 1 Medical College, Aga Khan University, Karachi, Pakistan; 2 Department of Surgery, Washington University in St. Louis, St. Louis, MO USA; 3 Department of Medicine, Aga Khan University, Karachi, Pakistan; 4 Department of Global Health and Social Medicine, Harvard Medical School, Boston, MA USA; 5 Department of Neurology, University of Toledo, Toledo, OH USA; 6 Department of Anaesthesiology, Aga Khan University, National Stadium Road, Karachi, Sindh 74800 Pakistan; 7 Department of Surgery, Aga Khan University, Karachi, Pakistan; 8 Department of Community Health Sciences, Aga Khan University, Karachi, Pakistan; 9 CITRIC Health Data Science Center, Aga Khan University, Karachi, Pakistan; 10 Institute of Global Health and Development, Aga Khan University, Karachi, Pakistan

## Abstract

**Background:**

In response to the COVID-19 pandemic, concerted efforts were made by provincial and federal governments to invest in critical care infrastructure and medical equipment to bridge the gap of resource-limitation in intensive care units (ICUs) across Pakistan. An initial step in creating a plan toward strengthening Pakistan’s baseline critical care capacity was to carry out a needs-assessment within the country to assess gaps and devise strategies for improving the quality of critical care facilities.

**Methods:**

To assess the baseline critical care capacity of Pakistan, we conducted a series of cross-sectional surveys of hospitals providing COVID-19 care across the country. These hospitals were pre-identified by the Health Services Academy (HSA), Pakistan. Surveys were administered via telephonic and on-site interviews and based on a unique checklist for assessing critical care units which was created from the Partners in Health 4S Framework, which is: Space, Staff, Stuff, and Systems. These components were scored, weighted equally, and then ranked into quartiles.

**Results:**

A total of 106 hospitals were surveyed, with the majority being in the public sector (71.7%) and in the metropolitan setting (56.6%). We found infrastructure, staffing, and systems lacking as only 19.8% of hospitals had negative pressure rooms and 44.4% had quarantine facilities for staff. Merely 36.8% of hospitals employed accredited intensivists and 54.8% of hospitals maintained an ideal nurse-to-patient ratio. 31.1% of hospitals did not have a staffing model, while 37.7% of hospitals did not have surge policies. On Chi-square analysis, statistically significant differences (*p* < 0.05) were noted between public and private sectors along with metropolitan versus rural settings in various elements. Almost all ranks showed significant disparity between public–private and metropolitan–rural settings, with private and metropolitan hospitals having a greater proportion in the 1st rank, while public and rural hospitals had a greater proportion in the lower ranks.

**Conclusion:**

Pakistan has an underdeveloped critical care network with significant inequity between public–private and metropolitan–rural strata. We hope for future resource allocation and capacity development projects for critical care in order to reduce these disparities.

**Supplementary Information:**

The online version contains supplementary material available at 10.1186/s13054-022-04046-5.

## Background

Critical care is a multidisciplinary specialty which encompasses the comprehensive diagnosis, management, and monitoring of patients who have, or are at risk of having, a life-threatening illness [1]. The requirements of an intensive care unit (ICU) are quite vast with the inclusion of an allocated location, interprofessional staff, specialized beds, and costly high-tech equipment [2]. The latter includes mechanical ventilators, oxygen and air supply ports, access to electricity, and adequate space for staff, patients, and attendees. Analogously, multidisciplinary staff including nurses, intensivists, and allied health professionals are the bastions of a functioning ICU with observational evidence suggesting that an abundance in staffing can be correlated with reduced ICU mortality and length of stay [3, 4].

The continued growth in global population and life expectancy has led to a rise in non-communicable disease, provoking an increase in the demand of intensive care units and resources [5, 6]. The advent of the COVID-19 pandemic in 2020 acutely increased that burden and revealed the global deficit in ICU capacity, with current estimates suggesting that at least 96 countries and territories have a density of less than 5.0 ICU beds per 100,000 population [7]. This gap is most evident in low–middle-income countries (LMIC), leading to a potential inability to manage the anticipated influx of critically ill patients in surge situations, like the COVID-19 pandemic [8–10].

The World Health Organization (WHO) advocates for well-defined systems of acute care for the critically ill and injured as an integral part of resilient national health care systems [11]. However, in order to successfully develop such systems in diverse LMIC settings, a thorough characterization and quantification of regional and national acute care capacity, human and material resources, and barriers to capacity growth are essential [11]. In a recent systematic review, it was found that only 15 of 36 low-income countries had any publishable data regarding their ICU capacity [8]. Due to this paucity of data, however, the capacity to care for critically ill patients in LMIC settings remains largely unknown. Where available, ICU beds are minimal (0–2.8 beds per 100,000 population), with most countries at around 2 per 100,000 including Pakistan [7, 8, 12–16].

To fill this knowledge gap, we evaluated critical care facilities across Pakistan by carrying out a national critical care resource assessment of infrastructure, equipment, and staffing. We aim to evaluate the capacity of Pakistan’s ICUs during the era of COVID-19 to outline shortcomings in care for critically ill patients. Our hope is to provide a comprehensive assessment in order to inform resource allocation and pandemic-planning by policy-makers, public–private partnerships, and other stakeholders in Pakistan’s healthcare system.

## Methodology

### Study objectives and design

Our survey assesses key central aspects required to deliver critical care safely and successfully to patients. By landscaping the ICU infrastructure across the country, we aimed to accentuate both the principal strengths and the gaps in our systems and practices, which would subsequently be then targeted with sustainable capacity building interventions. Our secondary objective is to establish a scoring and ranking system by which we can accurately assess critical care resources.

We conducted a nation-wide cross-sectional study to delineate the existing conditions of critical care facilities in Pakistan. The National ICU Preparedness Survey is an integral component of the overarching national scale-up endeavor, the COVID-19 Tele-ICU Project. This project is a key public–private collaboration between the Government of Pakistan and the Aga Khan University that aims to bolster critical care capacity and health care systems across the country.

### Study setting, population, and definitions

With over 220 million residents, Pakistan is a developing country that employs two large-scale independent healthcare networks: the public setup that is led by provincial governments and the private one that is administered by autonomous stakeholders. We defined ICU as any hospital unit with the availability of invasive mechanical ventilation for the maximum number of patients within that unit. We also surveyed high-dependency units (HDUs) if hospitals did not have ICUs available for the care of COVID-19 patients, and HDUs were defined as units which offered continuous monitoring and care to patients short of delivering invasive mechanical ventilation. Neonatal and pediatric facilities were excluded.

### Study instrument (Additional file 1)

We created a novel 52-point structured questionnaire with categorical responses based on the Partners in Health, 4S (Staff, Stuff, Systems, and Space) framework to assess and evaluate critical care facilities (Additional file 1) [17, 18]. The questionnaire was structured to account for contextual applicability after expert review.

This foundational assessment included information on essential and recommended facilities in an ICU/HDU. Basic hospital information collected included number of hospital beds, number of ICU beds and type of unit. The components were: Space (infrastructure), Staff (healthcare workers in a critical care unit) Stuff (consumable and non-consumable supplies, both medical and non-medical), and System (policies and protocols).

Each question was given a score of 1 if affirmative and 0 if not present or unknown. Unknown variables were captured but counted as not present for the purpose of analysis. The scores of each 4S component were then tallied. The Space component was scored out of 9, the Staff component out of 8, the Stuff component out of 19, and the System component out of 16.

### Data collection (Fig. 1)

**Fig. 1:**
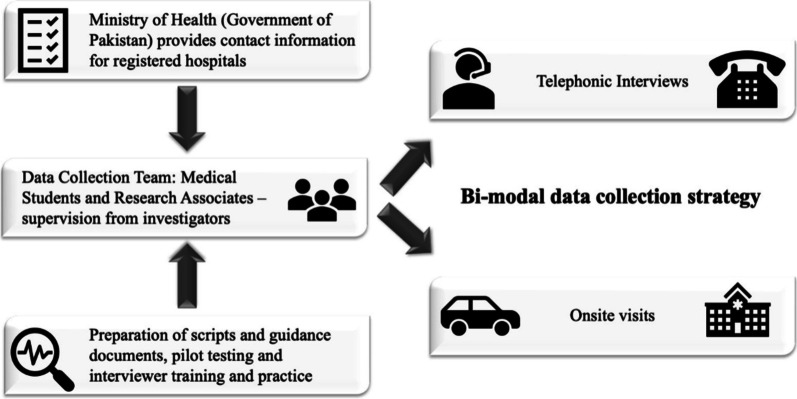
Data collection procedure

Data collection was done in two phases. In the first phase, the Pakistan Ministry of National Health Services Regulations and Coordination provided the core research team an official, authorized list of existing hospital facilities to be surveyed, along with their contact information. The first phase took place from May 2020 to November 2020. In the second phase, a list of hospitals was obtained from each of Pakistan’s respective provincial national ministries. This phase took place from June 2021 to August 2021. A bimodal strategy was executed, whereby telephonic or on-site interviews were conducted to complete the survey. Responses were obtained from healthcare workers primarily working in the critical care unit or administrative staff responsible for that unit. All collected data from the checklists were entered onto a standardized REDCap survey form.

### Quality control

Prior to data collection, all team members were trained through orientation and practice sessions. To further augment this and minimize inter-operator reporting bias, a guidance document with descriptions of each item on the checklist and a uniform written script were also provided to all interviewers. Pilot testing of the study instrument was done to validate it prior to official rollout of the survey. For this stage, we administered it to ICU leadership, including physicians and nurses at our home institution. Responses were verified through an in-person assessment by the study team and cross-referenced with formal hospital data. Phrasing of questions in the survey was adjusted accordingly to optimize clarity and ensure validity, while minimizing inter-rater variability. A central communications team was also established to keep track of all outgoing correspondence and conduct real-time troubleshooting. In order to account for the period of time between Phase 1 and Phase 2 of survey conduction, a sensitivity analysis was performed between the two sets of hospitals. Inter-rater variability was also measured between the hospitals which underwent different modes of surveys.

### Statistical analysis

Analyses were done using R version 4.1.1. Descriptive statistics have been reported as frequencies and percentages for the categorical variables and mean and standard deviation for the continuous variables. Chi-square analysis and Fischer’s exact test were done to analyze the differences between public against private hospitals, and also metropolitan against rural hospitals, with a p value of less than 0.05 considered significant. Responses were categorized as either yes or no, with unknown and missing being grouped together. Only definitive responses were included for analysis.

As the score of each of the 4S components did not add up to same amount, we used a weighted index by dividing the total number of questions by the number of questions in each component so that all the components were scored uniformly out of a denominator of 25. These sections were then also added up to make an overall percentage score out of 100. Analysis of variance (ANOVA) and post hoc Tukey’s honestly significant difference (HSD) tests were done to observe any statistically significant heterogeneity between component scores.

We undertook a cluster analysis to make group interpretation easier based on similarities in facility-level characteristics. The 4 different aspects of Space, Staff, Stuff, and System were considered, and hospitals were then ranked using the quartiles from the percentage scores. Ranks were also clustered within the individual components of Space, Staff, Stuff, and Systems, within which we observed the proportionate breakup of hospitals along the lines of hospital setting, sector, and size. These clusters were therefore indicative of how well resourced hospitals were in each particular component as well as overall. This grouping can allow stakeholders to broadly categorize which segment of a critical care facility requires improvements and to what degree.

Inter-rater variability was assessed for a subset of hospitals for whom both in-person and telephonic interviews were done. We used Cohen’s kappa statistic to compare the corresponding component scores of hospitals that were surveyed more than once. The kappa value was categorized using the framework outlined by Landis et al. [19]. A sensitivity analysis employing Chi-square and Fischer’s exact test was conducted between hospitals surveyed in Phase 1 and Phase 2 after matching for healthcare setting, sector, and hospital size.

### Ethical considerations and data management

This study was approved by the Ethical Review Committee, the institutional review board at the Aga Khan University. Verbal consent was obtained before every interview, and all data were kept confidential. Access to data was password-protected and limited to the core analysts only, to ensure due data privacy and security protocols.

## Results

A total of 135 hospitals were approached, of which 106 (78.5%) responded, and their characteristics are displayed in Table 1. Response rate for the capacity assessment questions was 99.31%, with only 38 missing variables. All these hospitals accepted care of adult COVID-19 patients. There were regional disparities in the distribution of critical care facilities, with almost 90% of ICUs/HDUs concentrated in Punjab, Sindh, and Khyber Pakhtunkhwa (KPK), respectively, and fewer facilities in Gilgit-Baltistan (5.7%), Azad Jammu and Kashmir (AJK) (4.7%), Baluchistan (0.9%) (Table 2). Seventy-six hospitals (71.7%) were in the public sector, 26 (24.4%) were private hospitals, and 4 (3.77%) were administrated by philanthropy-based foundations. Sixty hospitals (56.6%) were located in the metropolitan setting, while 46 (43.4%) were located in the rural setting.

**Table 1:** Hospital characteristics

Variables	Number of responses (*n* = 106)	Percentage
Provinces of Pakistan		
Punjab	33	31.13%
Sindh	30	28.30%
KPK	31	29.25%
AJK	5	4.72%
Baluchistan	1	0.94%
Gilgit-Baltistan	6	5.66%
Cities		
Metropolitan	60	56.60%
Non-Metropolitan	46	43.40%
Type of hospital		
Public	76	71.70%
Private	26	24.43%
Philanthropy-based	4	3.77%
Hospital size [number of beds]		
≤ 100	9	8.49%
100–499	56	52.83%
500–999	26	24.53%
≥ 1000	10	9.43%
Not reported (not known)	5	4.72%
Median	326	IQR = 200, 560
Stratification of critical care units included in the survey		
ICU	85	80.19%
HDU	12	11.32%
No critical care unit	9	8.49%
Average number of ICU beds†	12	IQR = 8,20
Cumulative number of ICU beds † (n = 86)	1560	
Average number of HDU beds†	9	IQR = 6,40
Cumulative number of HDU beds† (n = 39)	1105	
Type of respondent		
Consultant physician	47	44.34%
Trainee/medical officer	19	17.92%
ICU physician director	10	9.43%
Head nurse	7	6.60%
Administrative staff	11	10.38%
Staff nurse	4	3.77%
Others	8	7.55%

**Table 2:** Hospital capacity across Pakistan

	Number of beds	Number of ICU beds	Number of HDU beds	Ventilators	Population [20]
Total	51,592 (100%)	1560 (100%)	1105 (100%)	1185 (100%)	207,684,626
Punjab (*n* = 33)	23,100 (44.77%)	684 (43.85%)	152 (13.76%)	560 (47.26%)	112,019,014
Sindh (*n* = 30)	12,463 (24.16%)	515 (33.01%)	431 (39.00%)	273 (23.04%)	30,439,893
KPK (*n* = 31)	13,157 (25.50%)	262 (16.79%)	492 (44.52%)	287 (24.22%)	30,523,371
Balochistan (*n* = 1)	1062 (2.06%)	10 (6.41%)	20 (1.81%)	3 (0.25%)	12,344,408
Gilgit-Baltistan (*n* = 6)	410 (0.80%)	31 (1.99%)	0 (0.00%)	26 (2.19%)	1,249,000 (est.)
AJK (*n* = 5)	1400 (2.71%)	58 (3.71%)	10 (0.91%)	36 (3.04%)	4,045,366
Healthcare sector					
Public (*n* = 76)	43,495 (84.31%)	1145 (73.40%)	1075 (97.29%)	903 (76.20%)	
Private (*n* = 26)	6247 (12.11%)	333 (21.35%)	30 (2.71%)	253 (21.35%)	
Philanthropy-based (*n* = 4)	1850 (3.58%)	82 (5.26%)	0 (0.00%)	29 (2.45%)	
Healthcare setting					
Metro (*n* = 60)	36,378 (70.51%)	1136 (72.82%)	518 (46.88%)	834 (70.38%)	
Rural (*n* = 46)	15,214 (29.49%)	424 (27.20%)	587 (53.12%)	351 (29.62%)	

The mode of survey was largely telephonic, with 87 (82.1%) facilities being assessed over the phone. Eight (7.5%) surveys were done in-person, 4 (3.8%) were done over video software, and 7 (6.6%) were done through both physical and telephonic modes. Of the 29 hospitals which did not give consent, 19 (65.5%) were public, while 10 (34.5%) were private; 26 (89.7%) were location in a metropolitan setting, while 3 (10.3%) were located in rural areas. In terms of location, 16 (55.2%) were in Punjab, 9 (31.0%) in Sindh, 3 (10.3%) in Baluchistan, and 1 (3.5%) in Khyber Pakhtunkhwa.

Ninety-seven hospitals (91.5%) had either an ICU or HDU critical care facility. Of these 97 hospitals, 85 hospitals (80.2%) cared for COVID-19 patients in an ICU, while 12 (11.3%) only offered HDUs as the highest level of critical care support, while 9 (8.49%) hospitals did not have any COVID-19 critical care unit despite being listed as such in government registries. We included 86 ICUs and 39 HDUs overall as part of our survey which reported exact bed numbers, regardless of whether they cared for COVID-19 patients or not. The median number of total beds per facility was 326 (IQR = 360), ICU beds were 12 (IQR = 12), and HDU beds were 9 (IQR = 31). Survey respondents were traditionally consultant physicians (44.3%), followed by trainee medical officers (17.9%), and hospital administrative staff (10.4%).

Type of healthcare setup and geographical location were the main categories for comparison. Philanthropy-based facilities were included as private hospitals for the purpose of analysis. The number of ICU beds per hospital in the public sector is 15.1 beds, while in the private sector it is 13.8. In the metropolitan setting, it is 18.9, while in the rural setting, it is 9.2. There are 11.9 ventilators per hospital in public hospitals, 9.4 in private ones, 13.9 in metropolitan ones, and 7.6 in rural hospitals. Our 4S components were also analyzed along these lines.

### Space

The majority of units had gaps in their infrastructure and were not adequately equipped. Only 21 (19.8%) contained negative pressure rooms, with greater scarcity in public sector hospitals compared to private ones (*p* = 0.001). Fifty-nine facilities (55.6%) had no quarantine and lodging facility for the staff members, and isolation rooms were present in 74 facilities (69.8%). Significant difference was noted in the availability of medical air, vacuum, adequate gas, and adequate power outlets at the beds in public sector hospitals and rural areas as compared to private or metropolitan hospitals. Notably, rural areas are comparatively lacking in a centralized manifold for oxygen delivery (*p* = 0.048), with oxygen being delivered to patients via individual bedside cylinder. The mean score for the Space components was 5.91 out of a total of 9.

Detailed characteristics of the Space component can be seen in Table 3.

**Table 3:** Space component characteristics

Space	Total (*n* = 106)	Type of hospital			Healthcare setting		
		Public (*n* = 76)	Private (*n* = 30)	*P* value	Metropolitan (*n* = 60)	Rural (*n* = 46)	*P* value
Negative pressure rooms	21 (19.81%) [Obs = 104]	9 (11.84%) [Obs = 74]	12 (40.00%) [Obs = 30]	0.001	16 (26.67%) [Obs = 60]	5 (10.87%) [Obs = 44]	0.082
Isolation rooms/areas	74 (69.81%) [Obs = 98]	50 (66.67%) [Obs = 70]	24 (80.00%) [Obs = 28]	0.20	44 (73.33%) [Obs = 56]	30 (65.22%) [Obs = 42]	0.42
Adequate power outlets	81 (76.42%) [Obs = 103]	53 (70.67%) [Obs = 73]	28 (93.33%) [Obs = 30]	0.019	54 (90.00%) [Obs = 60]	27 (58.70%) [Obs = 43]	< 0.001
Adequate gas outlets	83 (78.30%) [Obs = 105]	55 (73.33%) [Obs = 76]	28 (93.33%) [Obs = 29]	0.006	54 (90.00%) [Obs = 60]	29 (63.04%) [Obs = 45]	0.001
Oxygen	86 (81.13%) [Obs = 105]	59 (78.67%) [Obs = 76]	27 (90.00%) [Obs = 29]	0.089	53 (88.33%) [Obs = 60]	33 (71.74%) [Obs = 45]	0.048
Medical air	75 (70.75%) [Obs = 104]	47 (62.67%) [Obs = 75]	28 (93.33%) [Obs = 29]	< 0.001	52 (86.67%) [Obs = 59]	22 (73.33%) [Obs = 45]	< 0.001
Vacuum	76 (71.70%) [Obs = 104]	48 (64.00%) [Obs = 75]	28 (93.33%) [Obs = 29]	< 0.001	52 (86.67%) [Obs = 60]	24 (52.17%) [Obs = 44]	< 0.001
Donning and doffing area	72 (67.92%) [Obs = 94]	51 (68.00%) [Obs = 68]	21 (70.00%) [Obs = 26]	0.55	41 (68.33%) [Obs = 54]	31 (67.39%) [Obs = 40]	0.86
Quarantine and lodging facility for staff members	59 (55.66%) [Obs = 80]	39 (52.00%) [Obs = 52]	20 (66.67%) [Obs = 28]	0.73	39 (65.00%) [Obs = 50]	20 (43.48%) [Obs = 30]	0.26

*Obs* number of definitive observations

### Staff

Most hospitals were well equipped with trainee doctors (95.2%) and assigned nurses (94.3%). However, 39 hospitals (36.8%) employed board certified intensivists, with significantly less prevalence in public (*p* = 0.001) and rural (*p* = 0.004) settings. Care of critical care patients was predominantly handled by anesthesiologists and pulmonologists. Similarly, despite the presence of nurses, only 58 ICUs (54.7%) featured the optimal nurse-to-patient ratio. The public sector and rural areas suffered from a significant dearth of sufficient nursing coverage (*p* < 0.001 for both). Access to pharmacists (68.9%), physical therapists (68.9%), and dedicated housekeeping staff (88.7%) was reasonable in all facilities. Very few hospitals had access to a dietician (35.9%), with significant decrease in availability in public (*p* < 0.001) and rural (*p* < 0.001) settings. The mean score for the Staff components was 5.43 out of a total of 8.

Detailed characteristics of the Staff component can be seen in Table 4.

**Table 4:** Staff component characteristics

Staff	Total (*n* = 106)	Type of hospital			Healthcare setting		
		Public (*n* = 76)	Private (*n* = 30)	*P* value	Metropolitan (*n* = 60)	Rural (*n* = 46)	*P* value
Availability of qualified intensivists	39 (36.79%) [Obs = 105]	21 (27.63%) [Obs = 76]	18 (60.00%) [Obs = 29]	0.001	29 (48.33%) [Obs = 59]	10 (21.74%) [Obs = 46]	0.004
Availability of trainee doctors/medical officers	101 (95.28%) [Obs = 105]	72 (94.74%) [Obs = 75]	29 (96.67%) [Obs = 30]	1.00	57 (95.00%) [Obs = 60]	44 (95.65%) [Obs = 45]	0.63
Nurses assigned to ICU							
Presence of nurses in ICU	100 (94.34%) [Obs = 100]	70 (92.11%) [Obs = 70]	30 (100.00%) [Obs = 30]	0.18	58 (96.67%) [Obs = 58]	42 (91.30%) [Obs = 42]	0.40
Availability of optimal nurse ratio (1:2/1:3)	58 (54.72%) [Obs = 99]	31 (40.79%) [Obs = 69]	27 (90.00%) [Obs = 30]	< 0.001	43 (71.67%) [Obs = 57]	15 (32.61%) [Obs = 42]	< 0.001
Ancillary staff/services							
Access to pharmacist	73 (68.87%) [Obs = 106]	51 (67.11%) [Obs = 76]	22 (73.33%) [Obs = 30]	0.53	44 (73.33%) [Obs = 60]	29 (63.04%) [Obs = 46]	0.26
Physical therapist	73 (68.87%) [Obs = 105]	48 (63.16%) [Obs = 75]	25 (83.33%) [Obs = 30]	0.052	44 (73.33%) [Obs = 60]	29 (63.04%) [Obs = 45]	0.33
Dietician	38 (35.85%) [Obs = 106]	18 (23.68%) [Obs = 76]	20 (66.67%) [Obs = 30]	< 0.001	30 (50.00%) [Obs = 60]	8 (17.39%) [Obs = 46]	< 0.001
Dedicated housekeeping/cleaning staff	94 (88.70%) [Obs = 104]	65 (85.52%) [Obs = 74]	29 (96.67%) [Obs = 30]	0.27	57 (95.00%) [Obs = 60]	37 (80.43%) [Obs = 44]	0.092

*Obs* number of definitive observations

### Stuff

Equipment was present in most facilities including ventilators (94.3%, mean = 11.97 ± 1.47) and BiPap machines (81.1%, mean = 9.48 ± 1.68), with a relative lack of high-flow nasal cannulas (56.6%, mean = 7 ± 1.52). However, there was significantly less ventilator availability in the rural setting (*p* = 0.005) and BiPap machine availability in public sector hospitals (*p* = 0.021). High-flow nasal cannulas were also significantly understocked in public hospitals (*p* = 0.034). Rural areas were also underserved in terms of the availability of intubation equipment (*p* = 0.005), vascular access devices (*p* = 0.003), and medication pumps (*p* = 0.047). Both public healthcare setups and rural facilities demonstrated a significant lack of information tools such as phones and computers, with public sector hospitals having additional limited internet availability. The mean score for the Stuff component was 16.00 out of a total of 19.

Detailed characteristics of the Stuff component can be seen in Table 5.

**Table 5:** Stuff component characteristics

Stuff	Total	Type of hospital (*n* = 106)			Healthcare setting (*n* = 106)		
	*n* = 106	Public (*n* = 76)	Private (*n* = 30)	*P* value	Metropolitan (*n* = 60)	Rural (*n* = 46)	*P* value
Personal protective equipment	99 (93.40%) [Obs = 106]	71 (93.42%) [Obs = 76]	28 (93.33%) [Obs = 30]	1.00	57 (95.00%) [Obs = 60]	42 (91.30%) [Obs = 46]	0.46
In-house laboratory testing facility	100 (94.34%) [Obs = 106]	70 (92.11%) [Obs = 76]	30 (100.00%) [Obs = 30]	0.18	57 (95.00%) [Obs = 60]	43 (93.48%) [Obs = 46]	1.00
Critical care drugs	98 (92.45%) [Obs = 106]	69 (90.79%) [Obs = 76]	29 (96.15%) [Obs = 30]	0.44	58 (96.67%) [Obs = 60]	40 (86.96%) [Obs = 46]	0.075
Vascular access devices	87 (82.08%) [Obs = 106]	58 (76.32%) [Obs = 76]	29 (96.67%) [Obs = 30]	0.012	55 (91.67%) [Obs = 60]	32 (69.57%) [Obs = 46]	0.003
Ventilators	100 (94.34%) [Obs = 106]	70 (92.11%) [Obs = 76]	30 (100.00%) [Obs = 30]	0.18	60 (100.00%) [Obs = 60]	40 (86.96%) [Obs = 46]	0.005
BiPap machine	86 (81.13%) [Obs = 105]	58 (76.32%) [Obs = 76]	28 (93.33%) [Obs = 39]	0.021	52 (86.67%) [Obs = 60]	34 (73.91%) [Obs = 45]	0.178
High-flow nasal cannula	60 (56.60%) [Obs = 105]	38 (50.00%) [Obs = 75]	22 (73.33%) [Obs = 30]	0.034	36 (60.00%) [Obs = 59]	24 (52.17%) [Obs = 46]	0.449
Integrated physiologic monitors	97 (91.51%) [Obs = 106]	68 (89.47%) [Obs = 76]	29 (96.67%) [Obs = 30]	0.44	59 (98.33%) [Obs = 60]	38 (82.61%) [Obs = 46]	0.010
Specialized beds for ICU/HDU patients	88 (83.02%) [Obs = 105]	61 (80.26%) [Obs = 75]	27 (90.00%) [Obs = 30]	0.38	53 (88.33%) [Obs = 59]	35 (76.09%) [Obs = 46]	0.058
Intubation equipment	100 (94.34%) [Obs = 106]	70 (92.11%) [Obs = 76]	30 (100.00%) [Obs = 30]	0.18	60 (100.00%) [Obs = 60]	40 (86.96%) [Obs = 46]	0.005
Medication pumps (for IVs, tube feed, etc.)	91 (85.85%) [Obs = 102]	61 (80.26%) [Obs = 72]	30 (100.00%) [Obs = 30]	0.031	57 (95.00%) [Obs = 60]	34 (73.91%) [Obs = 42]	0.047
Suction apparatus	103 (97.17%) [Obs = 106]	62 (81.58%) [Obs = 76]	30 (100.00%) [Obs = 30]	0.56	60 (100.00%) [Obs = 60]	43 (93.48%) [Obs = 46]	0.079
Crash cart with defibrillator	92 (86.19%) [Obs = 105]	54 (71.05%) [Obs = 75]	30 (100.00%) [Obs = 30]	0.018	55 (91.67%) [Obs = 60]	37 (80.43%) [Obs = 45]	0.15
X-ray machine	79 (75.96%) [Obs = 91]	69 (90.79%) [Obs = 65]	25 (83.33%) [Obs = 26]	0.17	47 (78.33%) [Obs = 53]	32 (69.56%) [Obs = 38]	0.53
Decontamination/cleaning materials and chemicals	97 (91.51%) [Obs = 106]	69 (90.79%) [Obs = 76]	28 (93.33%) [Obs = 30]	1.00	55 (91.67%) [Obs = 60]	42 (91.30%) [Obs = 46]	1.00
ICU patient information record/flow sheets	91 (85.85%) [Obs = 106]	63 (82.89%) [Obs = 76]	28 (93.33%) [Obs = 30]	0.22	51 (85.00%) [Obs = 60]	40 (86.96%) [Obs = 46]	0.77
Telephones	83 (78.30%) [Obs = 106]	55 (72.37%) [Obs = 76]	28 (93.33%) [Obs = 30]	0.019	53 (88.33%) [Obs = 60]	30 (65.22%) [Obs = 46]	0.004
Computers	71 (66.98%) [Obs = 106]	44 (57.89%) [Obs = 76]	27 (90.00%) [Obs = 30]	0.001	45 (75.00%) [Obs = 60]	26 (56.52%) [Obs = 46]	0.045
Internet connection	73 (68.87%) [Obs = 106]	46 (60.53%) [Obs = 76]	27 (90.00%) [Obs = 30]	0.003	45 (75.00%) [Obs = 60]	28 (60.87%) [Obs = 46]	0.12

*Obs* number of definitive observations

### System

Eighty-four hospitals (79.3%) had specific COVID-19 protocols in place. More than 80% of hospitals also had protocols in place for resuscitation, biomedical support, information technology (IT) support and transport. Fewer hospitals had such protocols for patient surge (62.3%), risk mitigation (51.9%), and environmental control (56.6%). Significantly fewer rural and public hospitals had support access via biomedical and IT services. Seventy-three hospitals (68.87%) had staffing models for doctors and nurses, but ICU workflow policies for nurses (*p* = 0.028) were reported to be significantly less in rural hospitals. Public sector hospitals showed gaps in emphasizing infrastructure failure (*p* = 0.003) and cardiopulmonary resuscitation (CPR) policy (*p* = 0.011), with rural hospitals also being less likely to implement CPR policies (*p* = 0.011) as well. The mean score for the System component was 11.68 out of a total of 16.

Detailed characteristics of the System component can be seen in Table 6.

**Table 6:** System component characteristics

System	Total (*n* = 106)	Type of hospital			Healthcare setting		
		Public (*n* = 76)	Private (*n* = 30)	*P* value	Metropolitan (*n* = 60)	Rural (*n* = 46)	*P* value
COVID management policy	84 (79.25%) [Obs = 105]	58 (76.32%) [Obs = 75]	26 (86.67%) [Obs = 30]	0.42	50 (83.33%) [Obs = 59]	34 (73.91%) [Obs = 46]	0.17
Staffing models for Doctors	73 (68.87%) [Obs = 101]	47 (61.84%) [Obs = 71]	26 (86.67%) [Obs = 30]	0.051	47 (78.33%) [Obs = 60]	26 (56.52%) [Obs = 41]	0.10
Staffing models for Nurses	73 (68.87%) [Obs = 101]	46 (60.53%) [Obs = 71]	27 (90.00%) [Obs = 30]	0.014	48 (80.00%) [Obs = 59]	25 (54.35%) [Obs = 42]	0.016
Admission policy	73 (68.87%) [Obs = 94]	49 (64.47%) [Obs = 67]	24 (80.00%) [Obs = 27]	0.11	46 (76.67%) [Obs = 54]	27 (58.70%) [Obs = 40]	0.042
Referral/discharge policy	75 (70.75%) [Obs = 94]	52 (68.42%) [Obs = 67]	23 (76.67%) [Obs = 27]	0.21	45 (75.00%) [Obs = 54]	30 (65.22%) [Obs = 40]	0.32
Surge policy	66 (62.26%) [Obs = 95]	45 (59.21%) [Obs = 69]	21 (70.00%) [Obs = 26]	0.14	40 (66.67%) [Obs = 56]	26 (56.52%) [Obs = 39]	0.62
Personal Protective Equipment policy	84 (79.25%) [Obs = 101]	57 (75.00%) [Obs = 73]	27 (90.00%) [Obs = 28]	0.035	50 (83.33%) [Obs = 58]	34 (73.91%) [Obs = 43]	0.34
CPR/Resuscitation policy	85 (80.19%) [Obs = 104]	56 (73.68%) [Obs = 74]	29 (96.67%) [Obs = 30]	0.011	54 (90.00%) [Obs = 60]	31 (67.39%) [Obs = 44]	0.011
Airway Management protocol	82 (77.36%) [Obs = 102]	55 (72.37%) [Obs = 72]	27 (90.00%) [Obs = 30]	0.17	53 (88.33%) [Obs = 59]	29 (63.04%) [Obs = 43]	0.005
Infrastructure failure policy	75 (70.75%) [Obs = 98]	48 (63.16%) [Obs = 70]	27 (90.00%) [Obs = 28]	0.003	49 (81.67%) [Obs = 59]	26 (56.52%) [Obs = 39]	0.061
Risk mitigation policy	55 (51.89%) [Obs = 82]	37 (48.68%) [Obs = 57]	18 (60.00%) [Obs = 25]	0.53	34 (56.67%) [Obs = 49]	21 (45.65%) [Obs = 33]	0.42
Environmental control policy	60 (56.60%) [Obs = 86]	39 (51.32%) [Obs = 60]	21 (70.00%) [Obs = 26]	0.14	38 (56.67%) [Obs = 53]	22 (47.83%) [Obs = 33]	0.62
Supply chain	80 (75.47%) [Obs = 94]	55 (72.37%) [Obs = 66]	25 (83.33%) [Obs = 28]	0.54	49 (81.67%) [Obs = 56]	31 (67.39%) [Obs = 38]	0.43
Biomedical support	95 (89.62%) [Obs = 106]	65 (85.52%) [Obs = 76]	30 (100.00%) [Obs = 30]	0.032	59 (98.33%) [Obs = 60]	36 (78.26%) [Obs = 46]	< 0.001
IT support	89 (83.96%) [Obs = 105]	60 (78.95%) [Obs = 76]	29 (96.67%) [Obs = 29]	0.005	56 (93.33%) [Obs = 60]	33 (71.74%) [Obs = 45]	0.005
Transport facility	89 (83.96%) [Obs = 106]	61 (80.26%) [Obs = 76]	28 (93.33%) [Obs = 30]	0.14	51 (85.00%) [Obs = 60]	38 (82.61%) [Obs = 46]	0.74

*Obs* number of definitive observations

### 4S scoring

We had hypothesized that private hospitals were better-resourced as compared to public ones and also that metropolitan hospitals more well equipped than rural ones. We performed a cluster analysis where we made 4 quartiles of ranks in each of the 4S components, and also in overall scoring. We then observed the breakdown of each rank in the components according to hospital setting, hospital sector, and hospital size in terms of bed numbers, and this breakdown is shown in Table 7, which shows statistically significant disparity between these strata.

**Table 7:** Proportionate ranks of the component scores

	Space				Staff				Stuff				System				Overall				
	Rank 1	Rank 2	Rank 3	Rank 4	Rank 1	Rank 2	Rank 3	Rank 4	Rank 1	Rank 2	Rank 3	Rank 4	Rank 1	Rank 2	Rank 3	Rank 4	Rank 1	Rank 2	Rank 3	Rank 4	
Hospital sector																					
Public	28	10	17	21	6	4	37	29	26	0	24	26	0	40	15	21	12	20	19	25	76
	63.6%	62.5%	73.9%	91.3%	33.3%	33.3%	88.1%	85.3%	54.2%	0.0%	77.4%	96.3%	0.0%	65.6%	68.2%	91.3%	44.4%	76.9%	73.1%	92.6%	
Private	16	6	6	2	12	8	5	5	22	0	7	1	0	21	7	2	15	6	7	2	30
	36.4%	37.5%	26.1%	8.7%	66.7%	66.7%	11.9%	14.7%	45.8%	0.0%	22.6%	3.7%	0.0%	34.4%	31.8%	8.7%	55.6%	23.1%	26.9%	7.4%	
*p* value	0.090				< 0.001				< 0.001				0.059				< 0.001				
Hospital setting																					
Metropolitan	31	10	14	5	16	10	22	12	38	0	12	10	0	41	11	8	23	16	13	8	60
	70.5%	62.5%	60.9%	21.7%	88.9%	83.3%	52.4%	35.3%	79.2%	0.0%	38.7%	37.%	0.0%	67.2%	50.0%	34.8%	85.2%	61.5%	50.0%	29.6%	
Rural	13	6	9	18	2	2	20	22	10	0	19	17	0	20	11	15	4	10	13	19	46
	29.5%	37.5%	39.1%	78.3%	11.1%	16.7%	47.6%	64.7%	20.8%	0.0%	61.3%	63.0%	0.0%	32.8%	50.0%	65.2%	14.8%	38.5%	50.0%	70.4%	
*p* value	0.002				< 0.001				0.001				0.025				< 0.001				
Hospital size																					
< 100	0	3	2	4	0	0	1	8	0	0	4	5	0	3	2	4	0	0	3	6	9
	0.0%	18.8%	8.7%	17.4%	0.0%	0.0%	2.4%	23.5%	0.0%	0.0%	12.9%	18.5%	0.0%	4.9%	9.1%	17.4%	0.0%	0.0%	11.5%	22.2%	
100–499	26	6	10	14	11	9	21	15	25	0	16	15	0	32	11	13	16	10	18	12	56
	59.1%	37.5%	43.5%	60.9%	61.1%	75.0%	50.0%	44.1%	52.1%	0.0%	51.6%	55.6%	0.0%	52.5%	50.0%	56.5%	59.3%	38.5%	69.2%	44.4%	
500–999	14	3	8	1	5	3	11	7	17	0	6	3	0	19	5	2	8	11	3	4	26
	31.8%	18.8%	34.8%	4.3%	27.8%	25.0%	26.2%	20.6%	35.4%	0.0%	19.4%	11.1%	0.0%	31.1%	22.7%	8.7%	29.6%	42.3%	11.5%	14.8%	
> 1000	4	3	2	1	2	0	8	0	5	0	4	1	0	7	1	2	3	4	2	1	10
	9.1%	18.8%	8.7%	4.3%	11.1%	0.0%	19.0%	0.0%	10.4%	0.0%	12.9%	3.7%	0.0%	11.5%	4.5%	8.7%	11.1%	15.4%	7.7%	3.7%	
Unknown	0	1	1	3	0	0	1	4	1	0	1	3	0	0	3	2	0	1	0	4	5
	0.0%	6.2%	4.3%	13.0%	0.0%	0.0%	2.4%	11.8%	2.1%	0.0%	3.2%	11.1%	0.0%	0.0%	13.6%	8.7%	0.0%	3.8%	0.0%	14.8%	
*p* value	0.004				0.008				0.014				0.033				0.002				
Total	44	16	23	23	48	12	31	27	18	12	42	34	0	61	22	23	27	26	26	27	106
	100%	100%	100%	100%	100%	100%	100%	100%	100%	100%	100%	100%	100%	100%	100%	100%	100%	100%	100%	100%	

Percentages are according to columns for the breakup in each rank

**Fig. 2:**
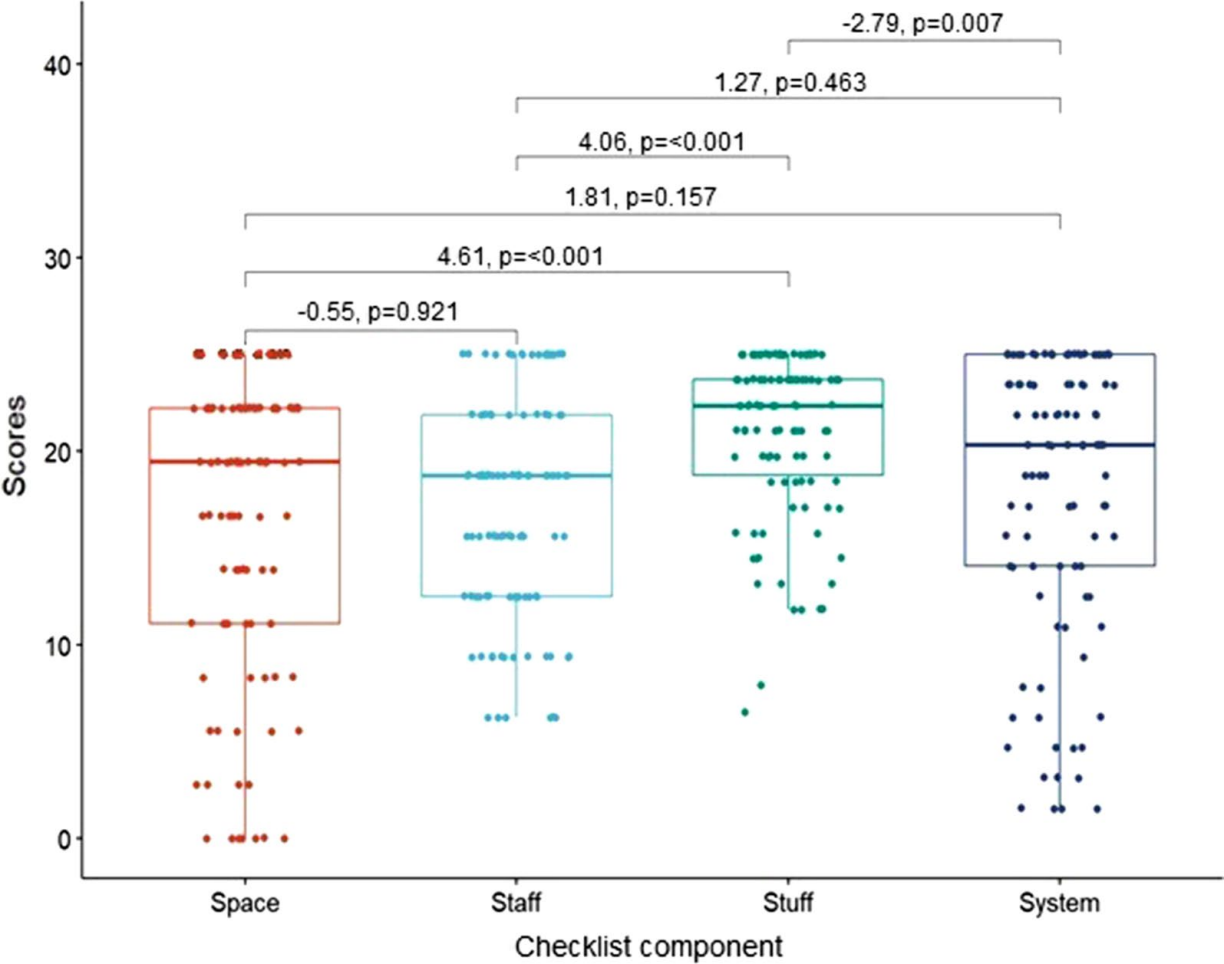
Tukey’s HSD testing of component scores, written as *n*, *p* value

ANOVA testing on the mean scores of each component yielded significant variation between the scores, F(3,424) = 11.2, *p* < 0.01. Tukey’s HSD post hoc comparisons were done between pairs of the 4 components and statistically significant differences were seen between Stuff–Staff (*p* < 0.001), Stuff–Space (*p* < 0.001), and System–Stuff (*p* = 0.008). Staff-Space (*p* = 0.921), System-Space (*p* = 0.157), and System-Staff (*p* = 0.463) did not show a statistically significant difference. The Stuff component had the highest mean weighted score. The results of this are presented in Fig. 2.

There were no hospitals in the 1st rank of the System component. In each component, and also overall, the majority of private hospitals scored in the 1st rank, with the exception of the System component where there were no hospitals in the 1st rank. A majority of metropolitan hospitals also scored in the 1st rank, except for in the Staff component, where a majority was seen in the 3rd rank, and in the System component where they were in 2nd rank. With the exception of the System component, hospitals in the 100–499 bed number range were consistently ranking 1st.

### Inter-rater variability

There were 7 facilities that were surveyed through both an in-person and a telephonic approach. Inter-rater variability was calculated between the 2 methods by observing the agreement for each question of the survey using Cohen’s kappa statistic. The calculated kappa was 0.86 (*p* value =  < 0.001), with 95.6% agreement versus an expected agreement of 68.5%. For non-congruent responses, we compared the proportion difference using z-score testing and found no statistically significant difference.

### Sensitivity analysis

A sensitivity analysis was carried out between hospitals whose data were collected in phase 1 and phase 2. Facilities were matched for healthcare sector, healthcare setting, and hospital size. We found that there was no significant improvement in critical care capacity among hospitals surveyed in Phase 2 compared to Phase 1. Hospitals in Phase 1 surveyed significantly better than those from Phase 2 in several domains, including 7 in the Space component, 3 in Staff, 11 in Stuff, and 16 in System. The largest differences were seen in the provision of a PPE policy, adequate power outlets, airway management protocol, and an established supply chain.

## Discussion

We found significant disparities between public/private and urban/rural hospitals with public and rural hospitals being significantly under-resourced. Overall, we found a deficiency in negative pressure rooms, qualified intensivists, nurses, and institutional policies across Pakistan. We also found that public sector hospitals and rural hospitals were significantly under-resourced in a number of areas. The novel scoring system we used to rank facilities offers a potentially promising approach for assessing health system capacity to care for critically ill patients.

Across the board, there is also a shortage of accredited intensivists and nurses in Pakistan’s critical care units. Only 36.79% of hospitals had even 1 qualified intensivist as the consultant physician in their ICU. While almost all hospitals employed nurses, only 54.72% of hospitals had an optimal nurse-to-patient ratio of 1:2, with a significant dip in their availability in both public and rural hospitals. The literature shows that higher physician-to-patient and nurse-to-patient ratios result in increased incidence of morbidity, mortality, and increased ventilator time for patients. Underqualification and understaffing could, therefore, lead to increased workload and compromised patient outcomes [3, 21–23]. Research to assess barriers toward critical care training is required to inform the advancement of accredited critical care training programs.

There were no hospitals at all that ranked 1st in our System component, showing that Pakistan’s ICUs require more well-defined organizational policies across the board. Several hospitals were lacking in protocols for admissions, surge situations, PPE, airway management, and infrastructure failure, which could compromise patient care. Pakistan’s public and rural hospitals were also significantly less likely to make use of staffing models for nurses. This could potentially leave critical care nurses more susceptible to burnout. Healthcare systems abroad employ tiered staffing models to circumvent shortages of healthcare workers by repurposing staff from other specialties for specific critical care procedures, and this is recommended in managing ICU surge capacity [24, 25]. More work should be done in introducing policies and strategizing around the current constraints in critical care human resources.

We observed substantial variation in the overall healthcare delivery of critical care units throughout Pakistan. Pakistan’s decentralized healthcare setup meant that we anticipated the differences in resources across provinces, as each province is responsible for the budgeting and upkeep of their own respective public hospitals. The lack of any robust healthcare coverage system means that substantial swathes of society are dependent on the subsidized public setup for healthcare. Therefore, the lack of adequate resources at these hospitals renders the less fortunate to inequitable critical care and possible morbidity or mortality [26, 27]. As of yet, there are no studies on the effect of public and private critical care on COVID-19 outcomes. However, there is the literature from Brazil, a high–middle-income country with a similar dichotomy in its public–private healthcare system as Pakistan, which showed that being treated at a public hospital ICU is an independent risk factor of mortality in sepsis patients [28]. They reported that these hospitals featured an “unfavorable patient-healthcare professional ratio, non-optimized processes, and a lack of adequate infrastructure”; these findings are also present in our setting.

Rural areas are more lacking in important consumable resources and infrastructural components, which is alarming because 63.56% of Pakistan’s population is based in rural areas, a sizeable majority [20]. They are lagging behind in several key characteristics in each section of our 4S checklist. While developed countries like the USA also experience disparity in critical care delivery between rural and metropolitan areas, the gap that we have found in our setting is more stark [29, 30]. The shortcomings in critical care delivery to these areas make its populace susceptible to the worst complications of critical COVID-19.

The current literature that we found on ICU capacity assessment only includes descriptive data. Our checklist scoring and clustering system represents a novel and potentially useful method of assessing hospital resources. ANOVA testing of the means of our component scores reveals that there is a significant difference between components, and we can provisionally say that our system of scoring and ranking hospitals is valid. It allowed us to see that critical care units in our setting are better equipped than previously thought, with Stuff having the highest mean score. However, in comparison with the relatively better supply of paraphernalia, ICUs and HDUs are significantly lagging behind in human resources, infrastructure, and hospital policies. Comparing clinical outcomes between hospital ranks could better help us assess our ranking system’s applicability.

There are some limitations to our study. The list of hospitals was obtained from government registries which meant that we did not have access to hospitals that were not featured on such registries. Balochistan was underrepresented, with only 1 hospital participating in our survey. In total, we were only able to identify 4 hospitals with ICUs from this province. More partnerships between the federal government and hospitals in Baluchistan are needed, as there was an overall lack of hospitals and limited accessibility to them. We were logistically unable to conduct a field visit at each hospital, which meant data collection was left to the knowledge of the telephonic respondents who may or may not have had an adequate inventory of their hospitals. However, an in-person survey on a sample of hospitals showed that there was substantial agreement. Finally, to address the fact that there was a lot of new learning that happened over time which may have affected COVID ICU care, we undertook a sensitivity analysis that showed no significant improvement in critical care capacity; initial impressions suggest that units surveyed in Phase 2 scored lower than those in Phase 1. This may be because hospitals surveyed in phase 2 were either smaller hospitals (e.g., secondary level hospitals), or from the smaller provinces that were added on at the request of individual ministries at a later date.

However, this is still the first national-level cross-sectional survey conducted during the COVID-19 era; it employs and adapts a validated framework to holistically assess and rank infrastructure, inventory, human resources, and protocols at each critical care unit. It can also be utilized for other capacity strengthening initiatives in Pakistan and worldwide [24, 31]. We have observed the disparities in Pakistan’s critical care delivery, between government and private hospitals and also between the metropolitan and rural settings. We hope that our study will encourage stakeholders to find targeted solutions to better critical care delivery across Pakistan such as training programs, broader investment, and creative thinking.

## Conclusion

The study has highlighted how Pakistan has an underdeveloped critical care network with significant inequity between across population densities and healthcare structure. The nature of Pakistan’s decentralized healthcare system and lacking infrastructure represent key areas for policy development and resource allocation by decision-makers to overcome the disparities in critical care. Our survey model may be replicated in other countries to assess the adequacy of healthcare delivery, in critical care and beyond.

## Supplementary Information

**Additional file 1.** Critical care capacity assessment tool.

## Data Availability

The datasets generated and/or analyzed during the current study are available from the corresponding author on reasonable request.

## Abbreviations

ICU: Intensive care unit
WHO: World Health Organization
LMIC: Low–middle-income country
HDU: High-dependency unit
ANOVA: Analysis of variance
Tukey’s HSD test: Tukey’s honestly significant difference test
Obs: Total number of definitive observations
IT: Information technology
CPR: Cardiopulmonary resuscitation
PPE: Personal protective equipment
