# Supplementary material for: Disparities in adult critical care resources across Pakistan: findings from a national survey and assessment using a novel scoring system
Source: Crit Care. 2022 Jul 11;26:209. doi: 10.1186/s13054-022-04046-5 (PMC9272593; doi:10.1186/s13054-022-04046-5)
Supplement: Supplementary file 1 — Additional file 1. Critical care capacity assessment tool. [file 13054_2022_4046_MOESM1_ESM.docx]

**Additional file 1**

**Critical care resources in Pakistan – findings from a national survey and assessment using a novel scoring system**

Record ID:

**PART 1 – FACILITY-LEVEL QUESTIONS**

Please fill in the following details for the hospital and respondent:

Please specify the type of interaction (Select all options that apply):

- Telephonic Interview
- Zoom Interview (Virtual Visit)
- Field Visit

Contact information of staff completing data collection (Name, phone, email):

Date of Data Collection (dd/mm/yy):

Location of Healthcare Facility Being Surveyed:

Healthcare facility name:

Healthcare facility region/district:

Healthcare facility address including city/town:

Location Type:

- Metropolitan/Urban
- Non-metropolitan/Rural
- Please specify the name of the city: ______________

Basic Information about the Healthcare Facility:

1. Which of the following terms best describes the healthcare facility?

- Government Hospital/Public Hospital
- Private Hospital
- Charity/NGO/Mission Hospital
- Others (Please specify): ______________

1. If you selected Government Hospital, please select the category/type:
   - Medical Teaching Institute (MTI)
   - Non-Medical Teaching Institute (Non-MTI)
   - District Headquarter Hospital (DHQ)
   - Tehsil Headquarter Hospital (THQ)
   - Rural Health Centers (RHCs)
2. How many total inpatient beds does this healthcare facility have?
   - <50
   - 50-99
   - 100-249
   - 250-499
   - 500-999
   - >999
   - Unknown

If available, please specify the total number of hospital beds: ___________________

1. Does this facility have an Intensive Care Unit (ICU)^[[1]](#footnote-1)^?
   - Yes
   - No
   - Unknown
2. How many ICU beds does this facility have that can provide continuous monitoring?
3. Does this facility have a High Dependency Unit (HDU)^[[2]](#footnote-2)^?
   - Yes
   - No
   - Unknown
4. How many HDU beds does this facility have that can provide continuous monitoring?
5. What is the overall number of consultant/attending physicians in this facility?
6. Respondent Profile
7. Please specify the type of respondent (multiple checkboxes):

- Consultant Physician
- Sub-specialty Physician
- ICU Physician Director/Head
- Trainee Physician/Medical Officer
- Head Nurse
- Staff Nurse
- ICU/HDU Technician
- Administrative Staff
- Others (Please Specify) _____________________________

1. For how many years has the respondent worked at this facility?

- <1 year
- 1-3 years
- >3 years
- Has never worked at this facility

Please type in any additional comments for this section:

**PART 2 – CRITICAL CARE UNIT QUESTIONS**

**PART 2A- GENERAL QUESTIONS**

1. Which of the following critical care units is being surveyed?

- ICU (with mechanical ventilator)
- HDU (without mechanical ventilator)
- Others

If you selected others, please specify: ________________________________

1. Please specify what is the specialty of the critical care unit being surveyed (multiple options can be selected)

- ICUs
- Adult ICU
  - General ICU
  - Medical ICU
  - Cardiac Critical Unit
  - Surgical ICU
  - Cardiac Surgical ICU
  - Neuro Critical Care Unit
  - Neurosurgical Unit
- Pediatric ICU
- Neonatal ICU
- HDU
  - Adult HDU
  - Pediatric HDU

1. Please specify the total number of beds in the unit which can provide continuous monitoring: ­­­­_________________

**PART 2B – SPACE (ICU/HDU COMPONENTS)**

Please specify whether the healthcare facility has the following components:

1. Negative Pressure Rooms
2. Do you have negative pressure rooms in this critical care unit?

- Yes
- No
- Unknown

1. Quantity Negative Pressure Rooms: ________
2. Isolation Rooms/Areas
   1. Do you have isolation rooms/areas in this critical care unit?

- Yes
- No
- Unknown

Quantity of Isolation Rooms: ________

1. Adequate Power Outlets
   1. Presence of Adequate Power Outlets^[[3]](#footnote-3)^ for each bed:
   - Yes
   - No
   - Unknown
   1. How often is electricity available?
      - Always (100%)
      - Almost always (76-99%)
      - Often (51-75%)
      - Sometimes (26-50%)
      - Rarely (1-25%)
      - Never (0%)
   2. Quantity of Power Outlets per bed: _____
2. Adequate Gas Outlets
3. Presence of Adequate Gas Outlets^[[4]](#footnote-4)^ per bed:
   1. Oxygen
   - Yes
   - No
   - Unknown
   1. Medical Air
   - Yes
   - No
   - Unknown
   1. Vacuum
   - Yes
   - No
   - Unknown

Quantity of Oxygen Gas Outlets per bed: ­­­­­_______

Quantity of Medical Air Gas Outlets per bed: ______

Quantity of Vacuum Gas Outlets per bed: ______

1. Dedicated Donning and Doffing Area
2. Is there a designated donning and doffing area in this unit?

- Yes
- No
- Unknown

1. Oxygen Supply System
2. How often is oxygen available?
   - - Always (100%)
     - Almost always (76-99%)
     - Often (51-75%)
     - Sometimes (26-50%)
     - Rarely (1-25%)
     - Never (0%)
3. Please specify the type of oxygen supply system?

- Individual bedside oxygen cylinder
- Central manifold with oxygen cylinders
- Central liquid oxygen tanks
- Individual oxygen concentrators
- Central oxygen concentrators

Please type in any additional comments for this section:

**PART 2C – STAFF (ICU/HDU COMPONENTS)**

How many of the following providers are currently employed by the healthcare facility:

1. Specialized Consultant Intensivists

1. Availability of specialized consultant intensivists^[[5]](#footnote-5)^:

- Yes
- No
- Unknown

1. How many consultant intensivists are available? ______________________
2. How much training experience do they have in the critical care setting?

- <6 months
- 6 months – 1 year
- 1-2 years
- >2 years

1. If specialized consultant intensivists are not available at your facility, please specify the sub-specialty of the attending physicians leading the critical care unit:

- Pulmonology
- Anesthesiology
- Internal Medicine
- Pediatrics
- Surgery
- Others, please specify: ___________

2. Trainee Doctors/Medical Officers

1. Availability of trainee doctors/medical officers in this unit:
   - Yes
   - No
   - Unknown

Number of trainee doctors/medical officers: ________

3. ICU Nurses

1. How many nurses work in this unit? ____________
2. On average, what is the approximate nursing provider-to-patient ratio in this critical care unit?

- 1:1-2
- 1:3-4
- 1:4-5
- 1:5-10
- 1:10
- NA

4. Ancillary Staff/Services:

1. Access to Pharmacist:
   - Yes
   - No
   - Unknown
2. Presence of Physical Therapist:
   - Yes
   - No
   - Unknown
3. Presence of Respiratory Therapist:
   - Yes
   - No
   - Unknown
4. Presence of Dietician:
   - Yes
   - No
   - Unknown
5. Presence of dedicated housekeeping/cleaning staff:
   - Yes
   - No
   - Unknown

Number of Pharmacists:

Number of Physical Therapists:

Number of Respiratory Therapists:

Number of Dieticians:

Number of housekeeping/cleaning staff:

Please type in any additional comments for this section:

**PART 2D – STUFF (ICU/HDU COMPONENTS)***

*Please see appendix with pictures of all the consumable and non-consumable medical equipment.

1. Please check the type of consumable medical supplies available in this unit:

| Consumable Medical Supplies | Please select the most suitable option: | | | |
| --- | --- | --- | --- | --- |
| Personal Protective Equipment | Always (100%) | Sometimes (50%) | Rarely (25%) | Never (0%) |
| In-house Laboratory Testing Facility in hospital | Always (100%) | Sometimes (50%) | Rarely (25%) | Never (0%) |
| Critical Care Drugs (eg, vasopressors, resurrectable drugs) | Always (100%) | Sometimes (50%) | Rarely (25%) | Never (0%) |
| Vascular Access Devices (Central, Arterial) | Always (100%) | Sometimes (50%) | Rarely (25%) | Never (0%) |
| Intubation Equipment (Laryngoscope) | Always (100%) | Sometimes (50%) | Rarely (25%) | Never (0%) |
| Suctioning Apparatus | Always (100%) | Sometimes (50%) | Rarely (25%) | Never (0%) |

2. Please check the type of non-consumable medical supplies available in this unit:

| Non-Consumable Medical Supplies | Please select the most suitable option | | |
| --- | --- | --- | --- |
| Ventilators | Yes | No | Unknown |
| Integrated bedside physiologic monitors | Yes | No | Unknown |
| ICU beds | Yes | No | Unknown |
| Medication Pumps | Yes | No | Unknown |
| Crash Cart with Defibrillator | Yes | No | Unknown |
| Portable X-ray Machines | Yes | No | Unknown |
| Specialized High flow nasal cannula devices | Yes | No | Unknown |
| BiPAP | Yes | No | Unknown |

Please specify the quantity for ventilators if possible:

Please specify the quantity for integrated bed-side physiologic monitors if possible:

Please specify the quantity for medication pumps if possible:

Please specify the quantity for crash cart with defibrillators if possible:

Please specify the quantity for portable x-ray machines if possible:

Please specify the quantity for high flow nasal cannulas if possible:

Please specify the quantity for high flow nasal cannulas if possible

Please specify the quantity for CPAP devices if possible:

Please specify the quantity for BiPAP devices if possible:

3. Please check the type of consumable non-medical supplies available in this unit:

| Consumable Non-Medical Supplies | Please select the most suitable option | | | |
| --- | --- | --- | --- | --- |
| Decontamination/ cleaning materials and chemicals | Always (100%) | Sometimes (50%) | Rarely (25%) | Never (0%) |
| Medical record paper/ flow-sheets | Always (100%) | Sometimes (50%) | Rarely (25%) | Never (0%) |

4. Please check the type of non-consumable non-medical supplies available in this unit:

| Non-consumable Non-Medical Supplies | Please select the most suitable option | | |
| --- | --- | --- | --- |
| Computers/Laptops | Yes | No | Unknown |
| Pagers | Yes | No | Unknown |
| Telephones | Yes | No | Unknown |

Please specify the quantity of computers/laptops in this unit:

Please specify the quantity of pagers in this unit:

Please specify the quantity of telephones in this unit:

How often are you able to access internet that is provided by this facility?

- Always (100%)
- Almost always (50%)
- Sometimes (25%)
- Never (0%)

Please type in any additional comments for this section:

**PART 2E – SYSTEMS (ICU/HDU COMPONENTS)**

Please specify the availability of the following protocols and policies in this unit:

1. Evidence-Based Management Guidelines

1. Do you have evidence-based management guidelines for Covid-19?

- Yes
- No
- Unknown

1. Staffing Models and Plans
2. Do you have staffing models and plans for doctors to help with planning and management?
   - Yes
   - No
   - Unknown
3. Do you have staffing models and plans for nurses to help with planning and management?
   - Yes
   - No
   - Unknown
4. Please specify whether the following patient-related policies are present in this unit:

| Patient-related policies | Please select the most suitable option | | |
| --- | --- | --- | --- |
| Admission Policy | Yes | No | Unknown |
| Referral/Discharge Policy | Yes | No | Unknown |
| Policies in case of surge (ie, triage, ventilator allocation) | Yes | No | Unknown |

1. Please specify whether the following institutional policies are present in this unit:

| Institutional Policies | Please select the most suitable option | | |
| --- | --- | --- | --- |
| CPR/ Resuscitation Protocol and Policy | Yes | No | Unknown |
| PPE policy | Yes | No | Unknown |
| Airway management protocols | Yes | No | Unknown |
| Policies in case of system failure | Yes | No | Unknown |
| Risk Mitigation Policy | Yes | No | Unknown |
| Environmental control and cleaning policy | Yes | No | Unknown |
| Established and proven supply chain for all consumables and non-consumables | Yes | No | Unknown |

6. Please specify whether this unit has the following support systems in place:

1. Bio-Medical Engineering support availability for equipment breakdown:

- Always (100%)
- Sometimes (50%)
- Rarely (25%)
- Never (0%)

1. Please specify whether this is available in-house or externally:

- In-house
- Externally
- Unknown

1. IT support availability for technical issues
   - Always (100%)
   - Sometimes (50%)
   - Rarely (25%)
   - Never (0%)
2. Please specify whether this is available in-house or externally:

- In-house
- Externally
- Unknown

7. Critical Care Transport Team:

1. Please specify whether a critical care transport team is available to help transport patients to different areas within the facility:

- Yes
- No
- Unknown

1. Please specify whether this is available for intra-facility transfers or inter-facility transfers:

- Intra-facility transfer
- Inter-facility transfer

Please type in any additional comments for this section:

**PART 2F – APPENDIX**

1. Pictures of Infrastructural Components

- Negative Pressure Rooms


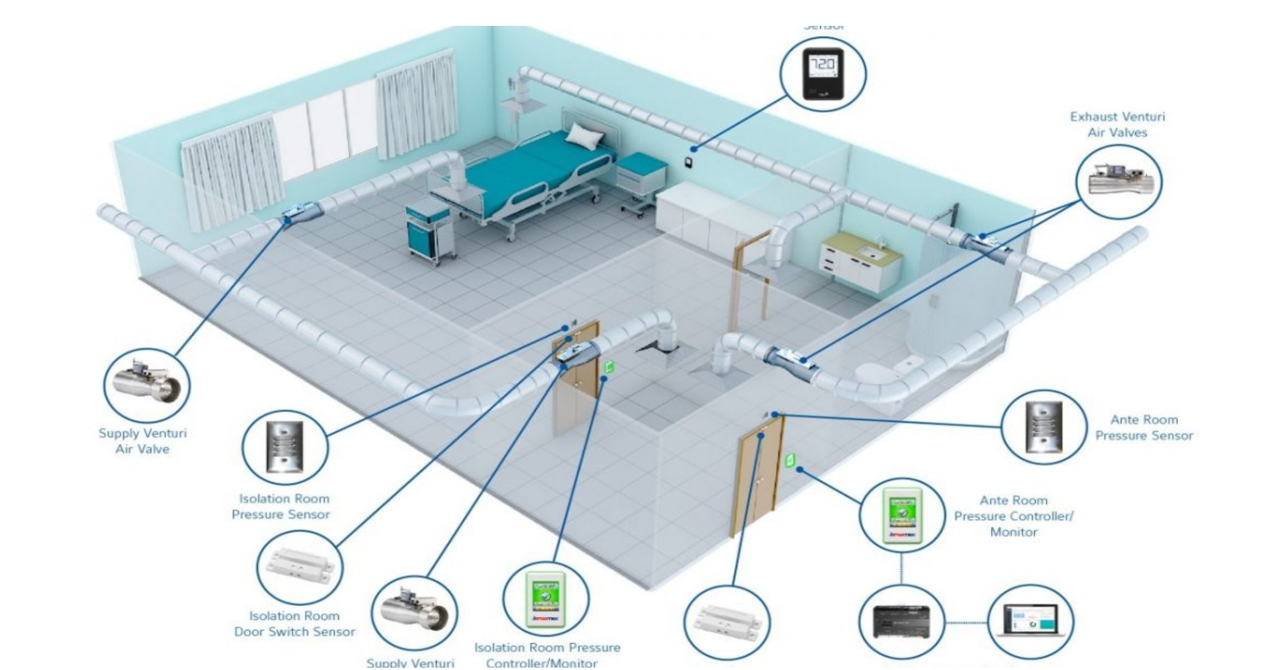


- Isolation Rooms
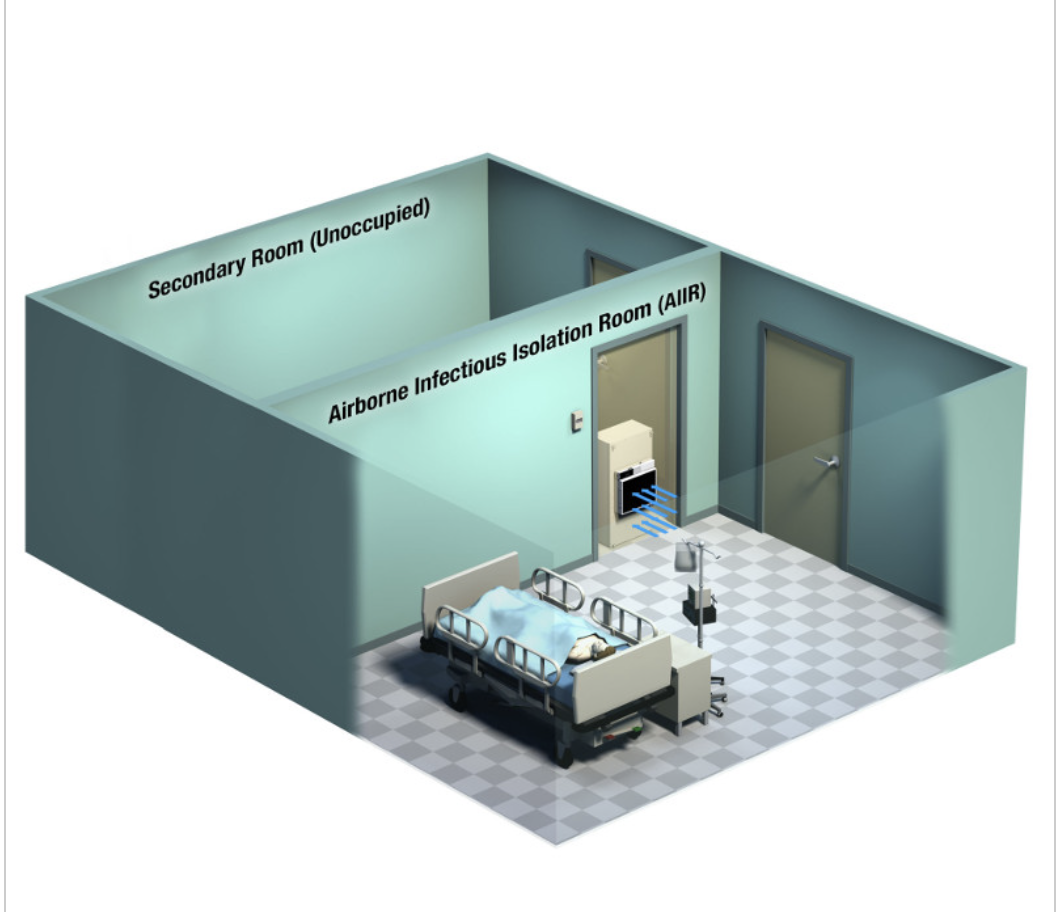

- Power Outlets


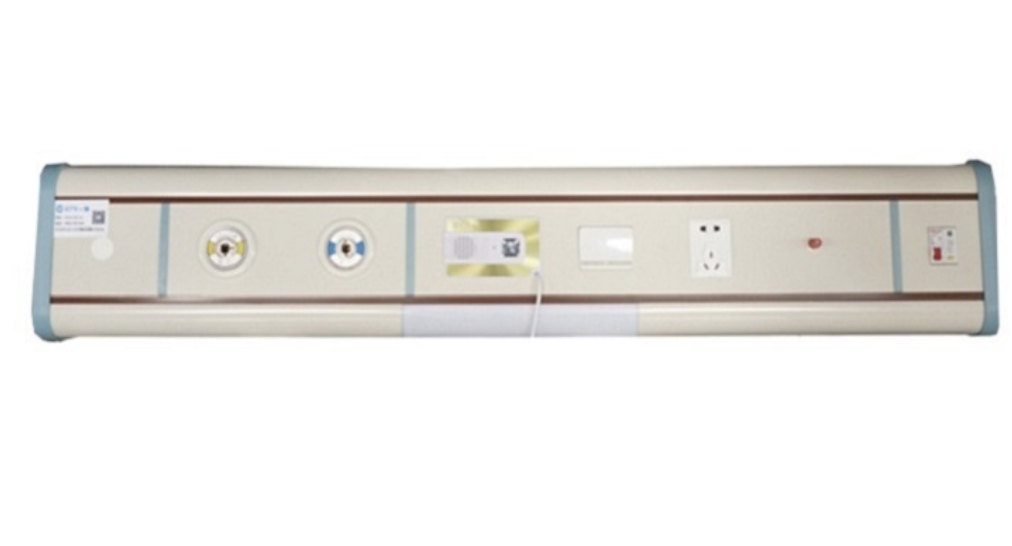


- Gas Outlets (Oxygen, Medical Air and Vacuum)


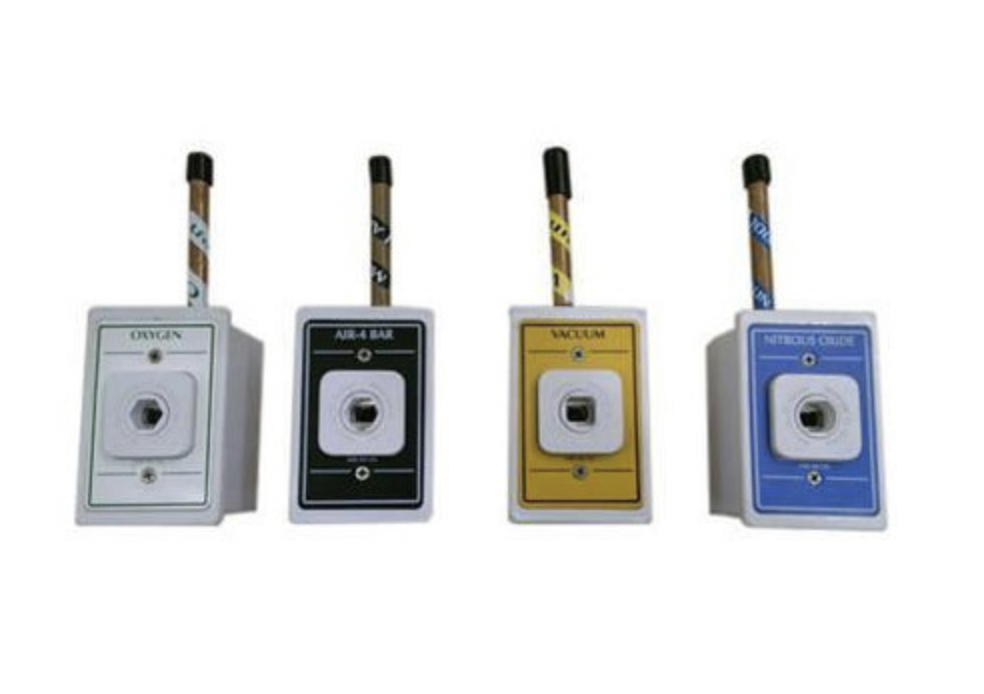


- Oxygen Supply System:
- Oxygen Concentrator


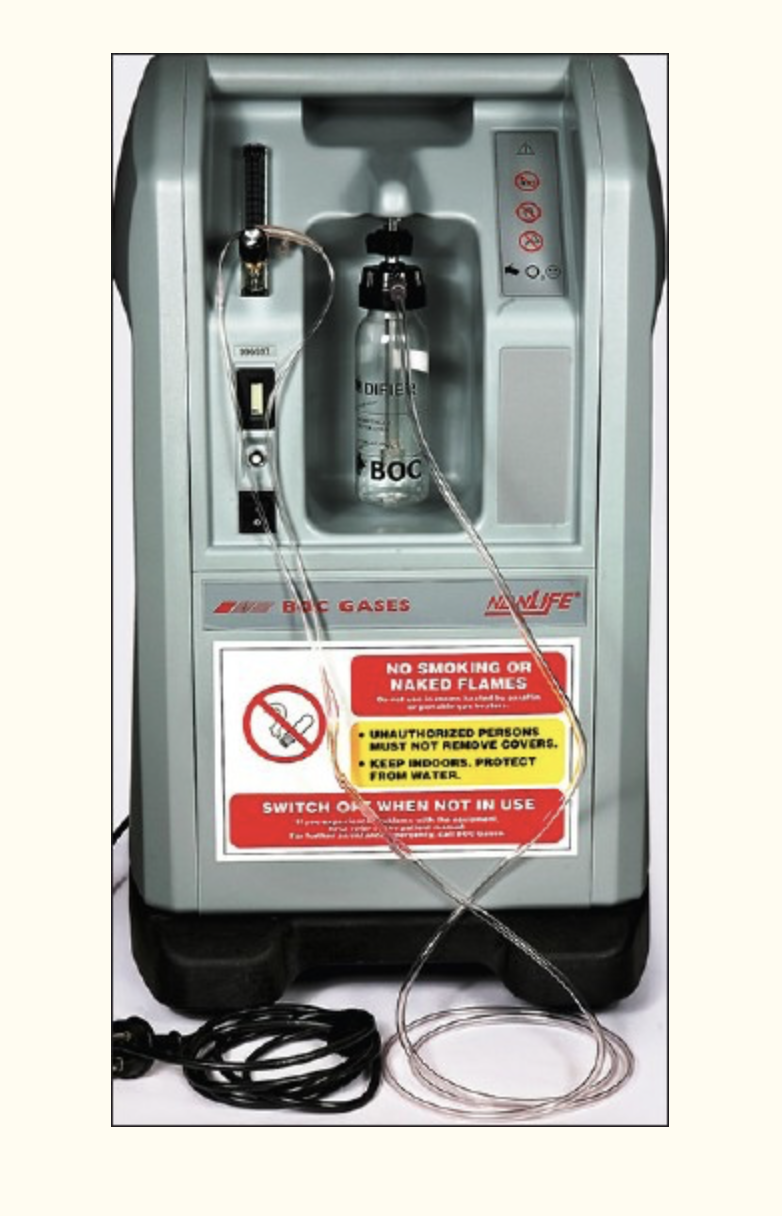


- -
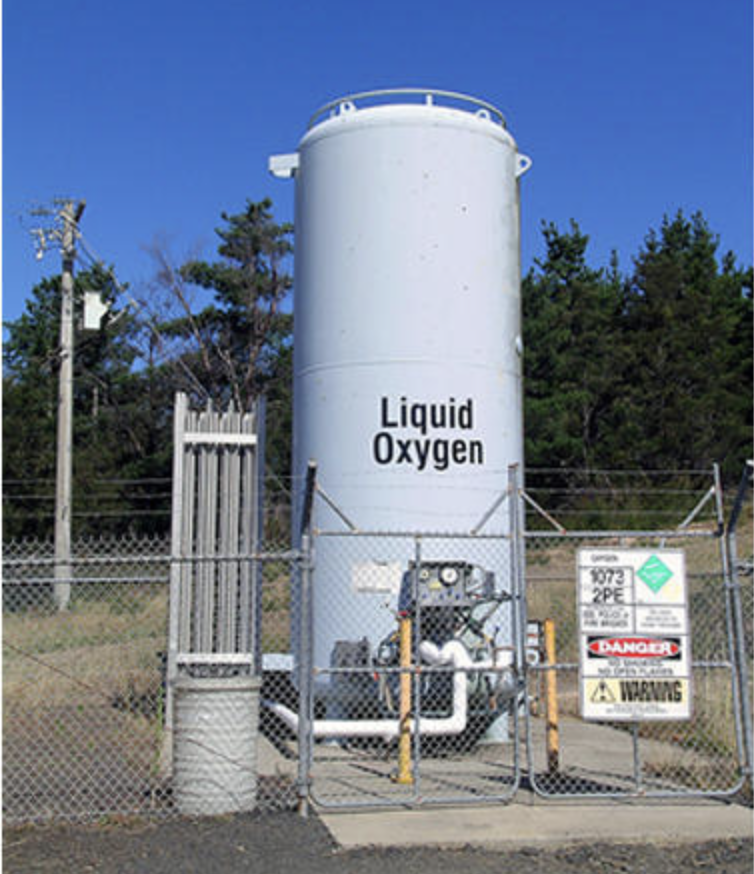
Liquid Oxygen Tanks
- Cylinder Manifold


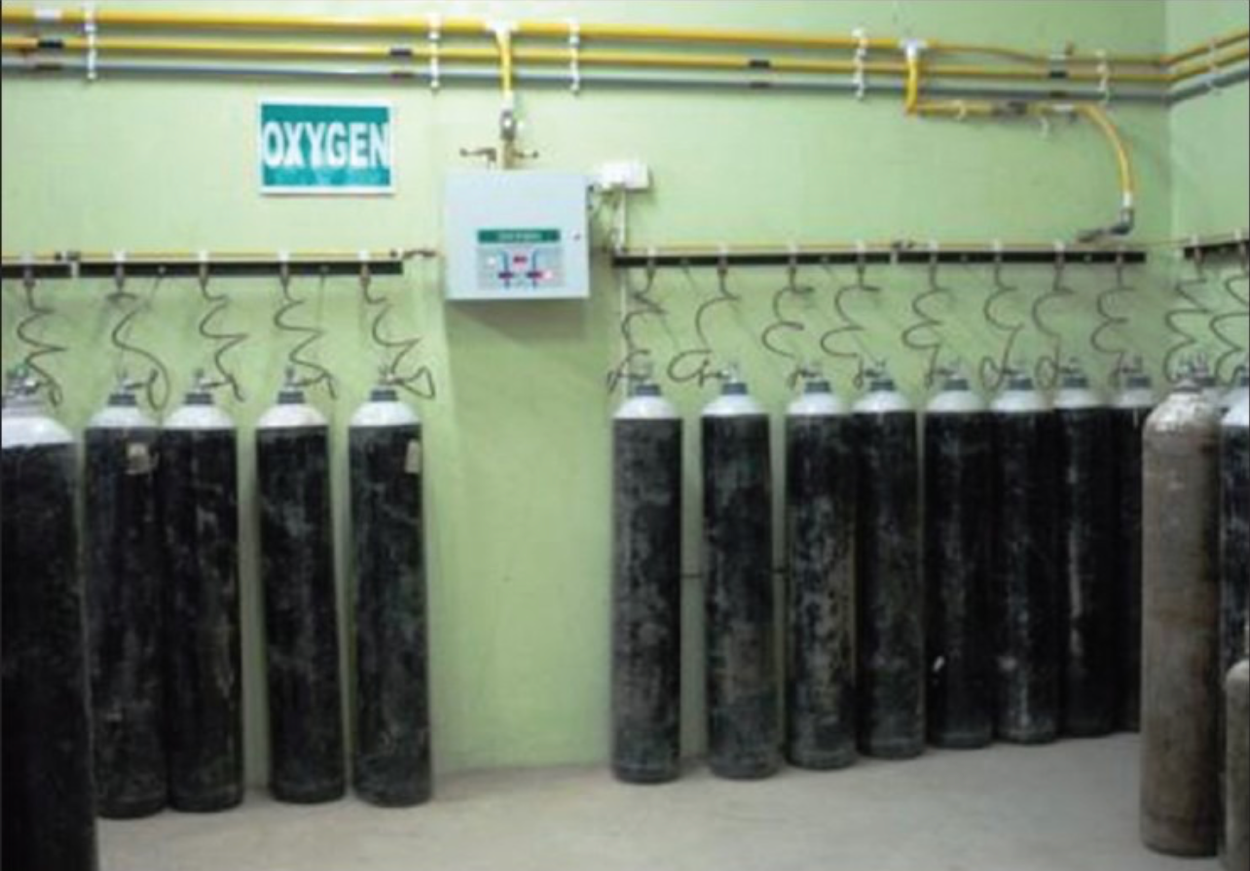


1. Pictures of Consumable Medical Supplies

- Vascular Access Device (Central):


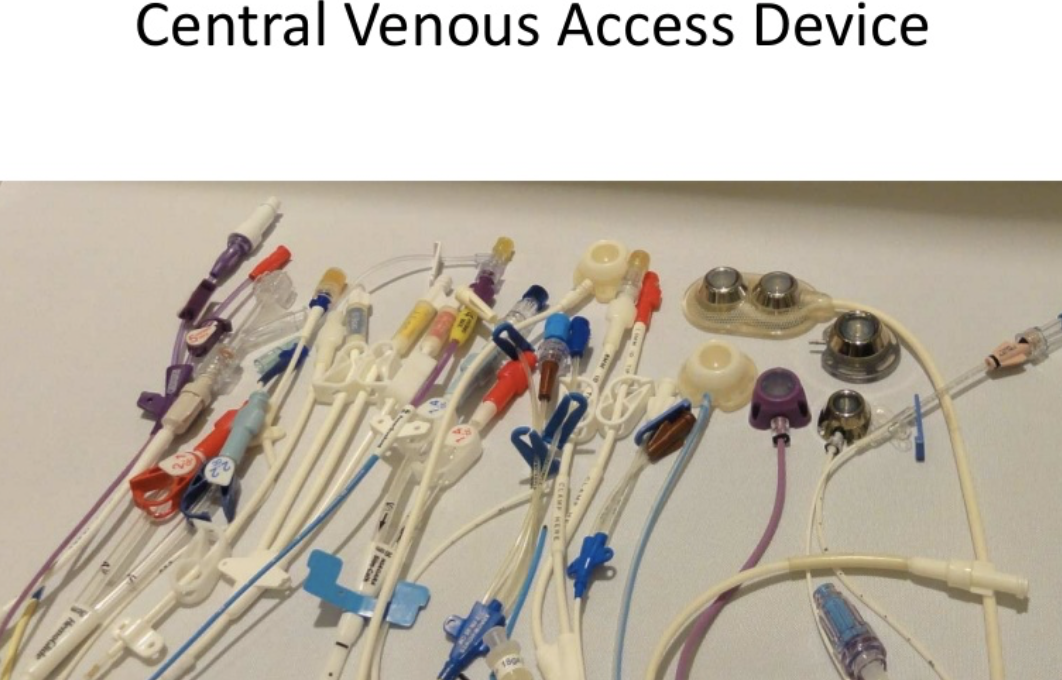


- Vascular Access Device (Peripheral):


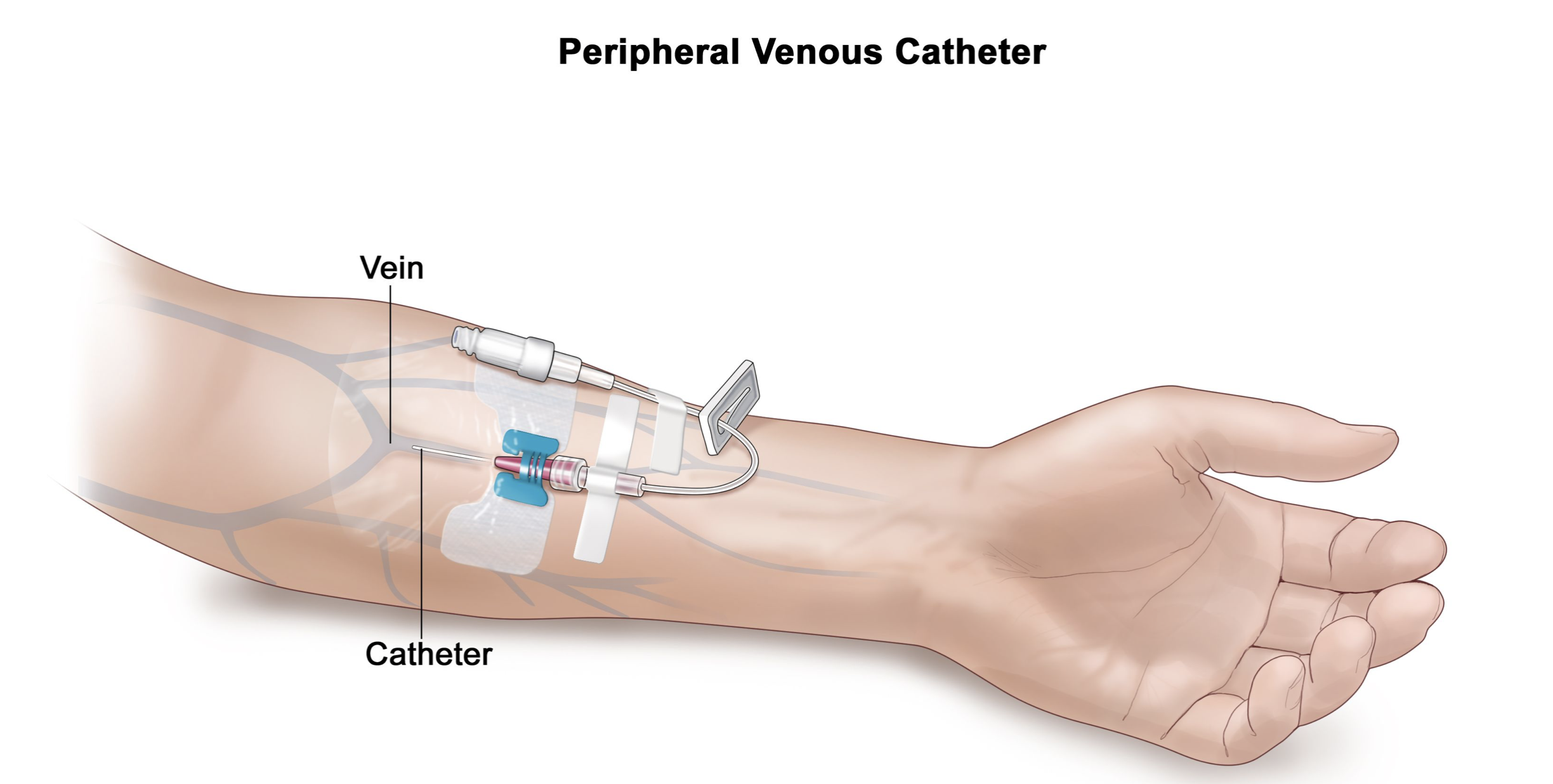


- Monitoring equipment for A-line/CVP line pressures:


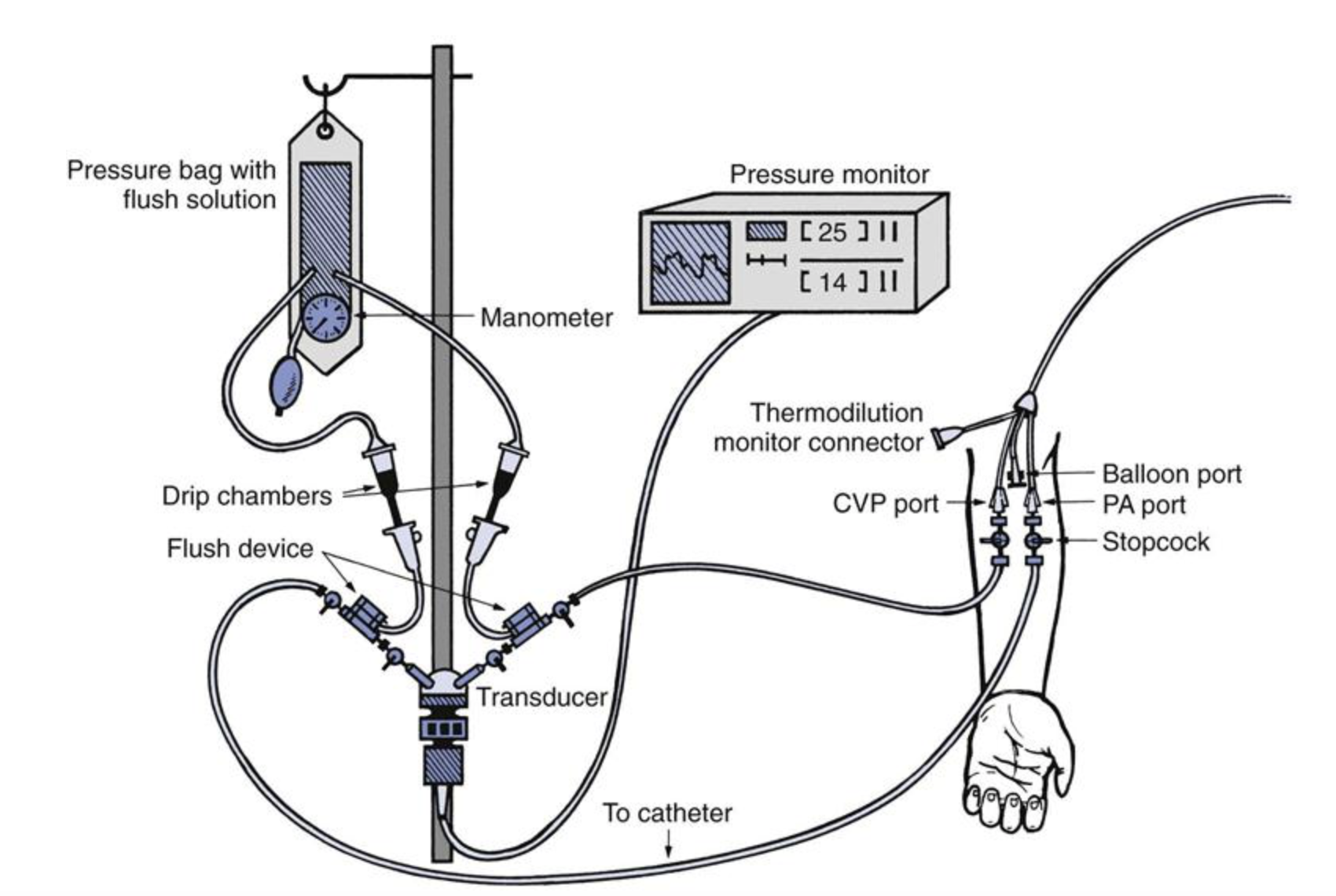


- Intubation Equipment (eg, Laryngoscopes)


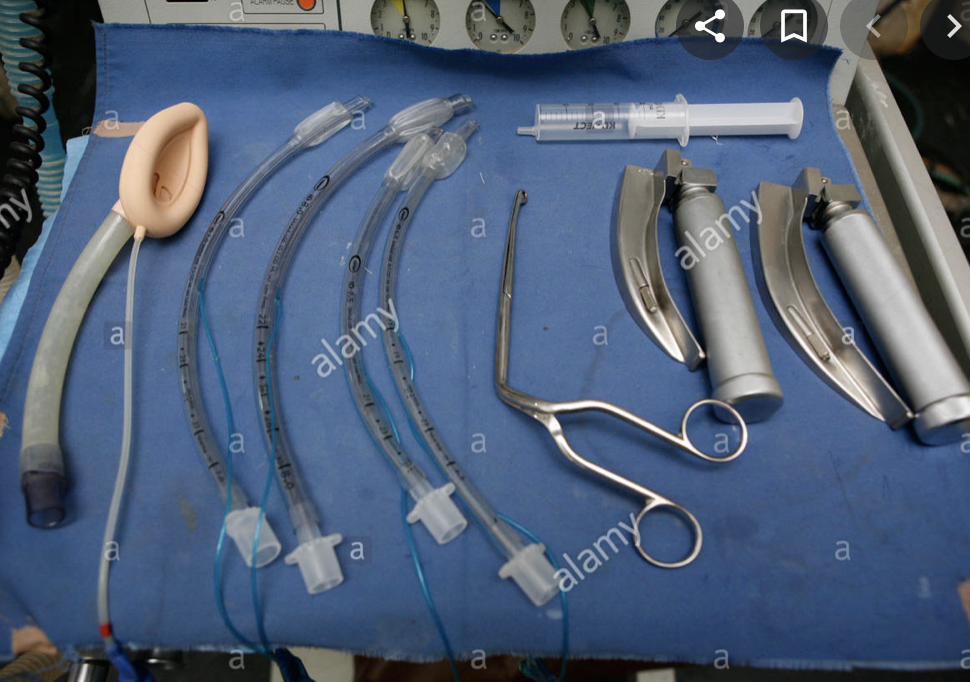


- Suction Equipment:


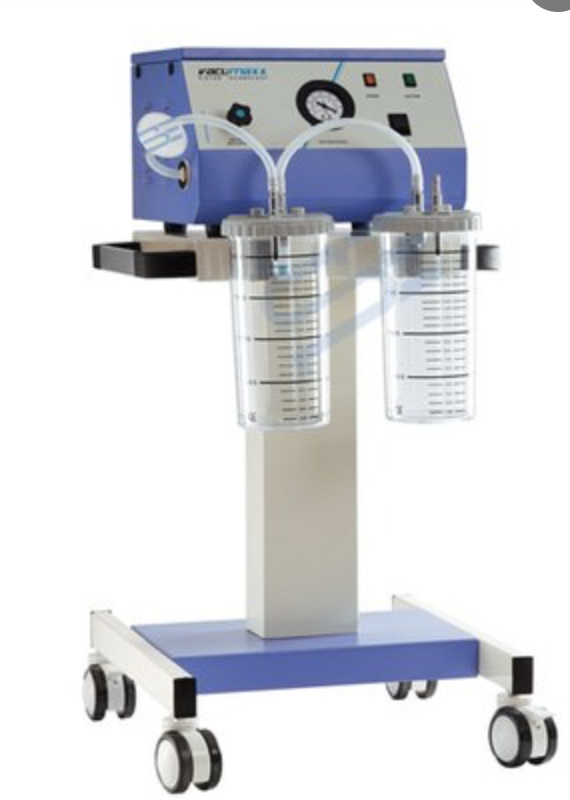


1. Pictures of Non- Consumable Medical Supplies

- Ventilators


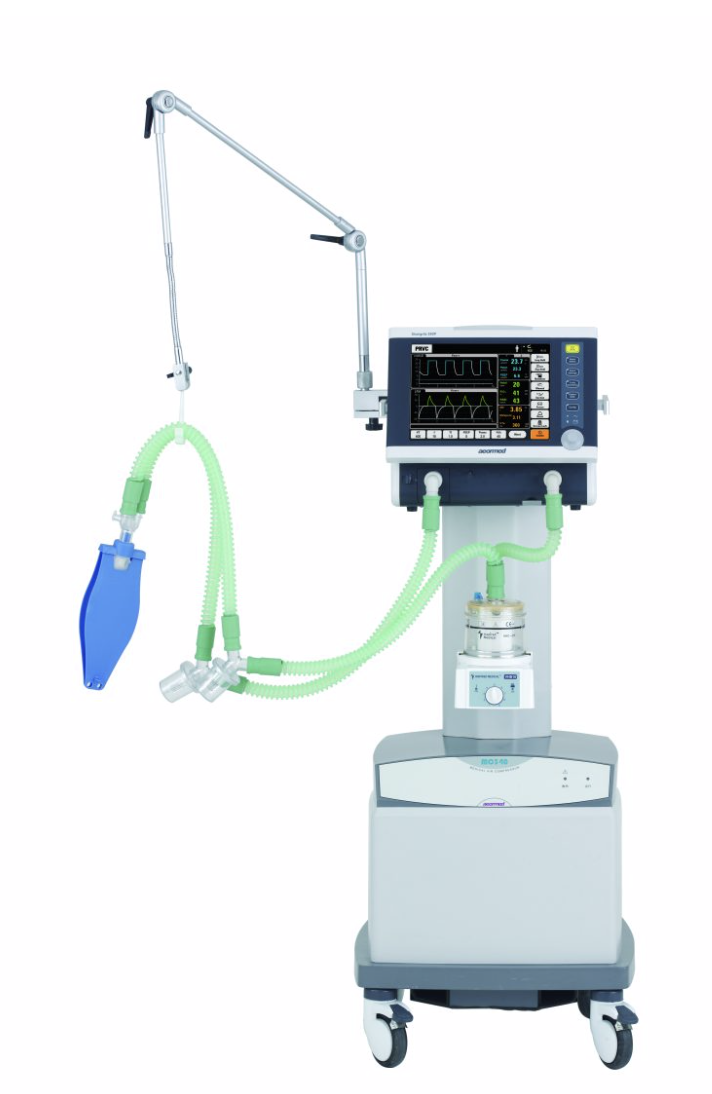


- Integrated bedside physiologic monitors


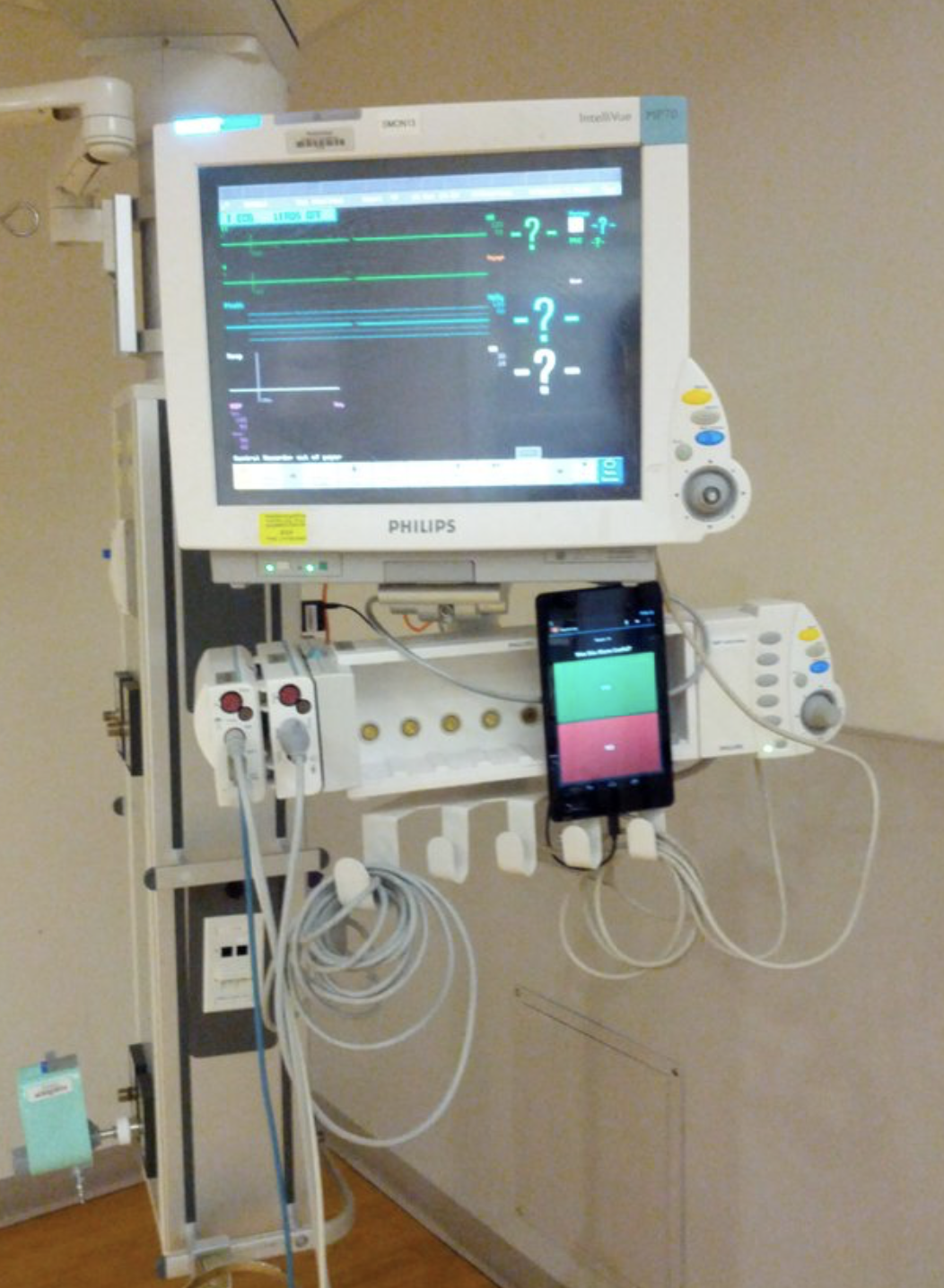


- ICU bed


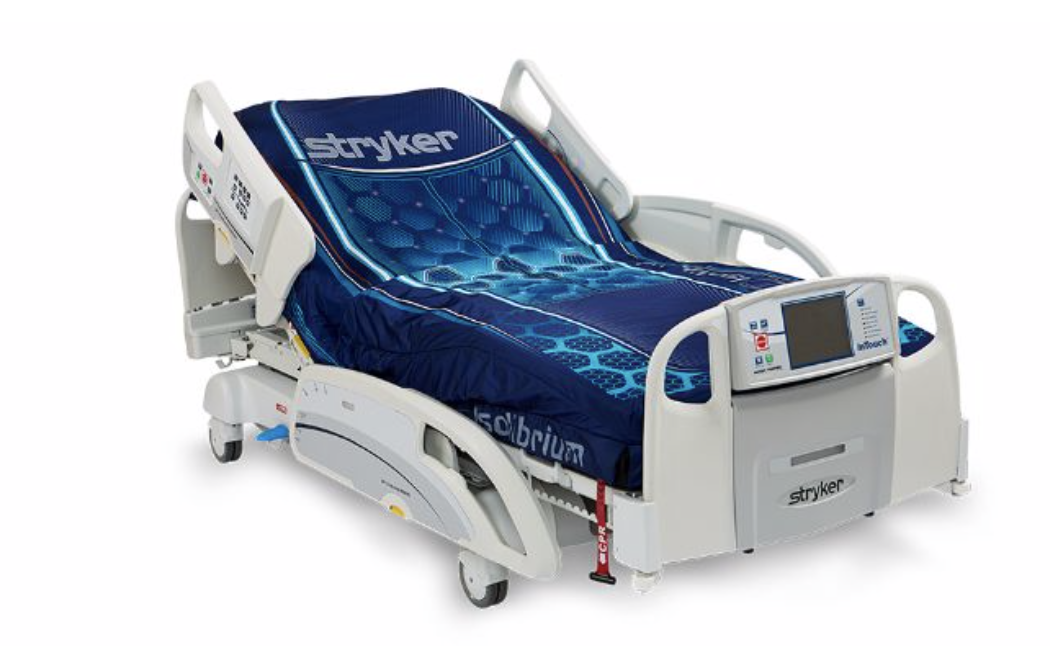


- Medication pumps


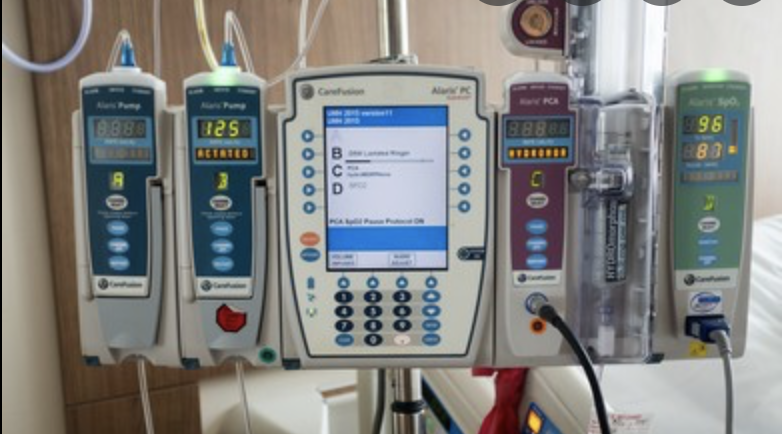


- Crash cart with defibrillator


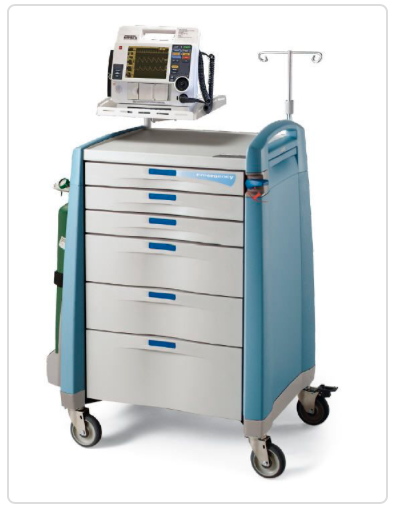


- Portable Ultrasound Machine


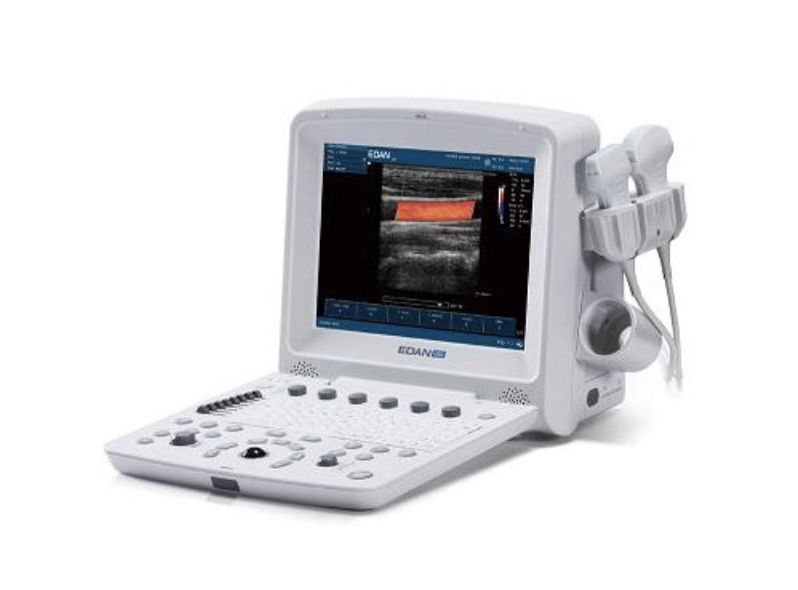


- Portable X-ray Machine


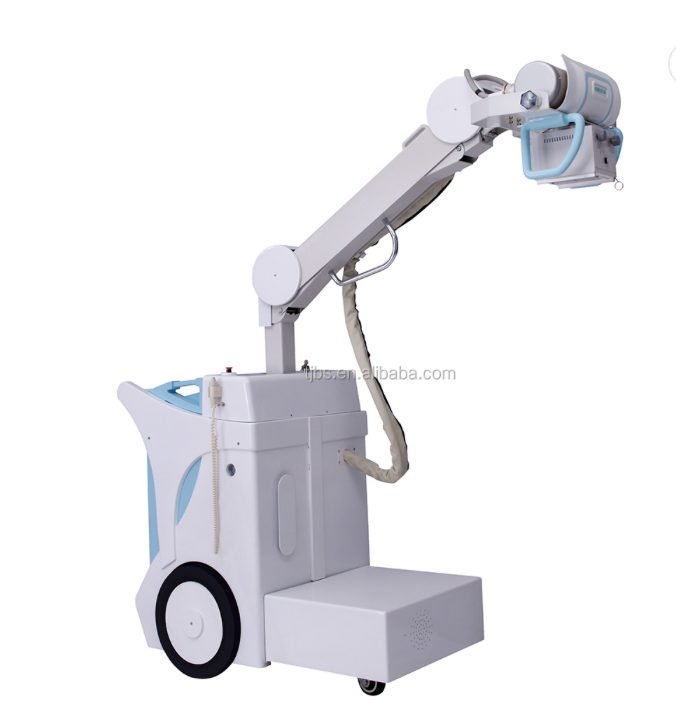


- Specialized High-flow nasal cannula devices


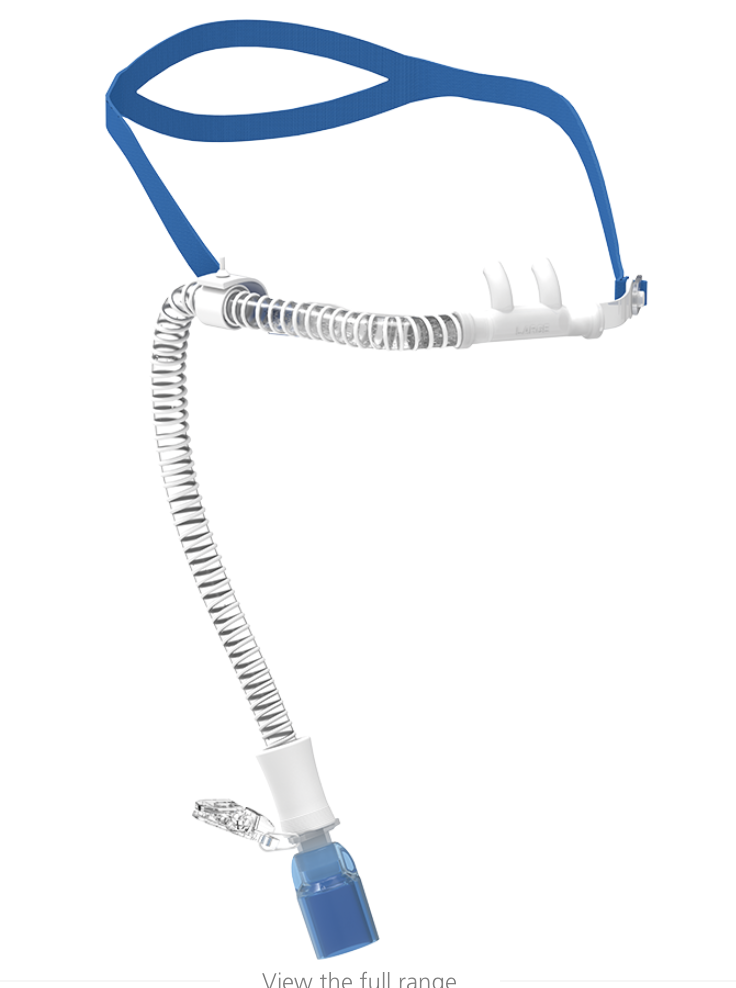


- Non-invasive ventilation (ie, CPAP and BiPAP machines)


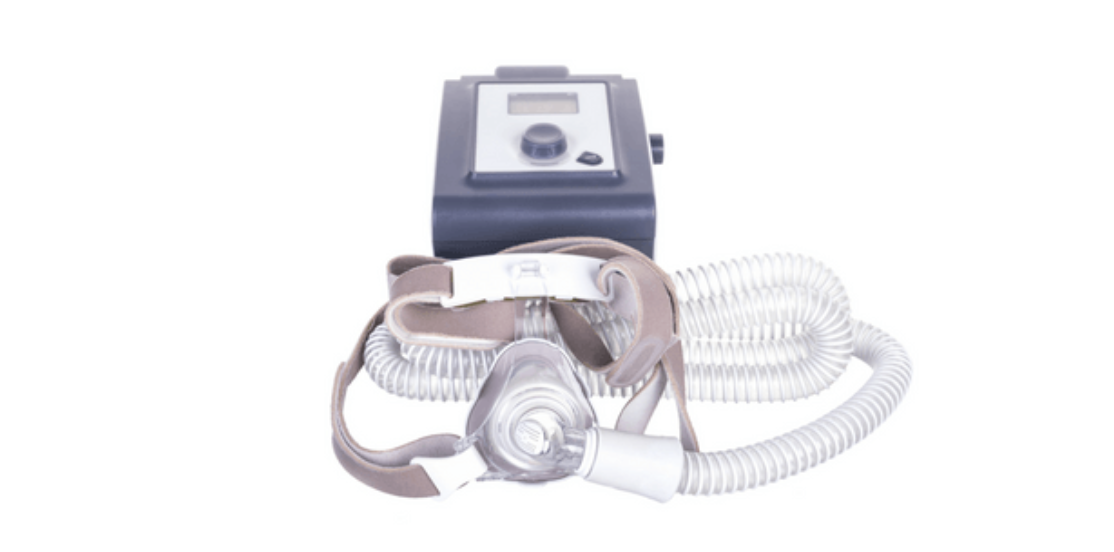


- Warmers and incubators


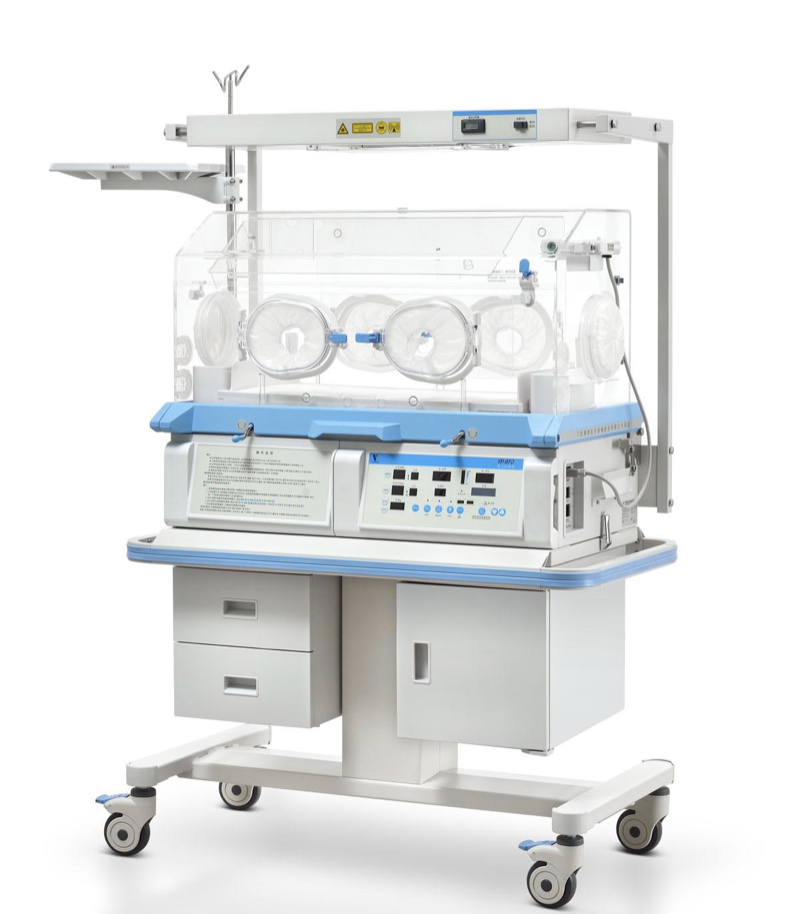


1. ICU - Intensive Care Unit: An intensive care unit (ICU) is an organized system for the provision of care to critically ill patients that provides intensive and specialized medical and nursing care, an enhanced capacity for monitoring, and multiple modalities of physiologic organ support to sustain life during a period of acute organ system insufficiency. [↑](#footnote-ref-1)
2. HDU - High Dependency Unit: An HDU is a specially staffed and equipped area of a hospital that provides a level of care intermediate between intensive care and the general ward care. It provides invasive monitoring and support for patients with or at risk of developing acute (or acute-on-chronic) single-organ failure, particularly where the predicted risk of clinical deterioration is high or unknown. It may act as a ‘step-up’ or ‘step-down’ unit between the level of care delivered on a general ward and that in an ICU. [↑](#footnote-ref-2)
3. Adequate Power Outlets: Ideally should include 28 power outlets per bed, with a standardized rating (voltage supply) and an ability to work continuously. [↑](#footnote-ref-3)
4. Adequate gas outlets: Ideally should include a minimum of 4 oxygen outlets, 2 medical air outlets and 2 vacuum gas outlets per bed, but numbers can vary depending on the facility. [↑](#footnote-ref-4)
5. Intensivist: An intensivist is a consultant physician responsible for coordinating and providing integrated care to the patient with acute and chronic complex illnesses in the critical care setting, who has been trained and certified through a primary specialty and has successfully completed an ACGME or board certified training program in critical care medicine and/or has a certificate of special qualification in critical care. [↑](#footnote-ref-5)
